# Aflatoxin B1-Induced Apoptosis in Donkey Kidney via EndoG-Mediated Endoplasmic Reticulum Stress

**DOI:** 10.3390/vetsci12020130

**Published:** 2025-02-05

**Authors:** Yanfei Ji, Yu Zhang, Wenxuan Si, Jing Guo, Guiqin Liu, Changfa Wang, Muhammad Zahoor Khan, Xia Zhao, Wenqiang Liu

**Affiliations:** College of Agriculture and Biology, Liaocheng University, Liaocheng 252000, China

**Keywords:** AFB1, donkey, kidney, nephrotoxicity, EndoG, endoplasmic reticulum stress, apoptosis

## Abstract

Aflatoxin B1 (AFB1) is a prevalent environmental and forage contaminant that poses significant health risks to both humans and livestock due to its toxic effects on various organs and systems. Among its toxicological effects, nephrotoxicity is a hallmark of AFB1 exposure. However, the precise mechanisms underlying AFB1-induced kidney damage in donkeys remain poorly understood. To investigate this, we established a donkey model exposed to AFB1 by administering a diet supplemented with 1 mg AFB1/kg for 30 days. Kidney apoptosis was assessed using TUNEL staining, while gene expression and protein levels of Endonuclease G (EndoG), as well as genes related to endoplasmic reticulum (ER) stress and apoptosis, were quantified by RT-qPCR and Western blotting. Our findings indicate that AFB1 exposure resulted in significant kidney injury, apoptosis, and oxidative stress. Notably, AFB1 exposure upregulated the expression of EndoG and promoted its translocation to the ER, which subsequently induced ER stress and activated the mitochondrial apoptotic pathway. These results suggest that AFB1-induced kidney damage in donkeys is mediated through the oxidative stress and mitochondrial apoptosis pathways, primarily involving the EndoG-IRE1/ATF6-CHOP signaling axis.

## Simple Summary

This study investigates the effects of Aflatoxin B1 (AFB1) on kidney damage in donkeys, aiming to uncover the molecular mechanisms and pathways responsible for nephrotoxicity. Aflatoxin B1 is a common environmental contaminant known to pose significant health risks to both humans and livestock, with nephrotoxicity being one of its most prominent toxicological effects. In this study, a donkey model was exposed to AFB1, resulting in observable kidney damage, apoptosis, and oxidative stress. The findings highlight the upregulation of Endonuclease G (EndoG), which plays a crucial role in triggering endoplasmic reticulum (ER) stress and activating mitochondrial apoptosis. These results provide valuable insights into the molecular mechanisms underlying AFB1-induced nephrotoxicity in donkeys.

## 1. Introduction

Aflatoxin is a type of mycotoxin primarily produced by Aspergillus flavus and Aspergillus parasiticus, with different chemotypes that includes aflatoxin B1 (AFB1), B2, B2a, G1, G2, G2a, M1, and M2. Among these, AFB1 is considered the most toxic and highly carcinogenic [1,2,3]. AFB1 is commonly found in the environment, soil, food crops, and animal feed. The consumption of contaminated feed can lead to livestock disease and even death [4,5]. Over 80% of ingested AFB1 is absorbed in the duodenum and jejunum through passive transport. It then accumulates in the liver and kidneys. The metabolites of AFB1 are primarily excreted through the kidneys, resulting in a relatively high residual amount of AFB1 in the kidneys. This can cause renal dysfunction and damage [6]. Research has reported that oxidative stress is a common and significant mechanism in the toxicology of AFB1 [7]. AFB1 disrupts the antioxidant system in mammalian cells and induces the overproduction of reactive oxygen species (ROS), which triggers oxidative stress damage and other signaling cascades, ultimately resulting in cell death [8,9,10]. Studies have also shown that chronic AFB1 intoxication can cause various injuries in livestock and poultry, including anorexia, liver necrosis, gallbladder enlargement, and intestinal congestion. These injuries occur through multiple biological processes, such as inflammation, apoptosis, and programmed death [11,12,13,14,15]. Additionally, AFB1 can cause renal injury by activating oxidative stress-related signaling pathways and apoptosis [16].

**Figure 1:**
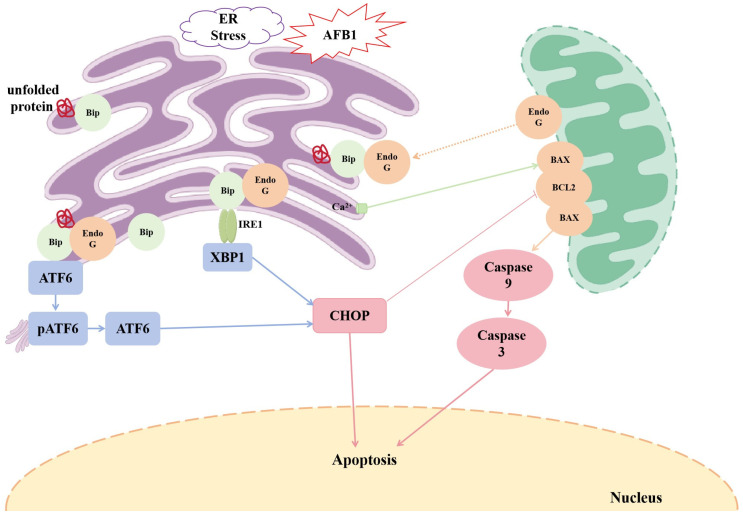
Schematic diagram of the AFB1-induced endoplasmic reticulum stress-related pathway.

Endonuclease G (EndoG) is a nuclear-coded protein located in the intermembrane space of mitochondria. When subjected to external stimulation, EndoG can translocate to the endoplasmic reticulum (ER) and bind to Bip (also named GRP78), resulting in the release of IRE1a/PERK and activation of the ER stress response [17]. The ER plays a crucial role in cellular protein fold and quality control and Ca^2+^ storage and release, which are essential for cell survival [18]. Protein folding and quality control rely on ER-associated degradation (ERAD) and unfolded protein response (UPR). UPR is a feedback control system that acts as a self-protection mechanism in ER stress through detecting unfolded protein pressure in the ER lumen through intracellular signaling, and three pathways are involved, namely ATF6, IRE1, and PERK [19,20]. Note that the ER provides an oxidative environment for protein folding, and protein oxidation increases ROS levels, leading to oxidative stress [21]. It has been demonstrated that prolonged ER stress leads to apoptosis, initiated by the IRE1-XBP1 pathway that enhances the transcriptional expression of the molecular chaperone protein C/EBP CCAAT enhancer binding protein (CHOP), the key factor of apoptosis induced by ER stress, to promote apoptosis [22]. Moreover, ATF6 migrates to the nucleus in response to nuclear localization signals and induces the expression of CHOP [23]. In addition, the disruption of ER calcium homeostasis leads to cytoplasmic Ca^2+^ overload, resulting in the activation of calpain, activation of the mitochondrial pathway, and the caspase cascade triggering apoptosis [24]. ER stress and the pro-apoptotic effects of the UPR pathway contribute largely to kidney injury. The mechanism of ER stress-related pathways induced by AFB1 is presented in Figure 1.

The contamination of animal feed with AFB1 is a significant threat to animal health. Different livestock species have varying susceptibility to AFB1, and donkeys are more sensitive to AFB1 compared with ruminants due to their monogastric nature and their use of the cecum for plant fiber digestion [5]. Therefore, an AFB1 exposure donkey foal model has been established in this study to investigate the nephrotoxic effects of AFB1 on donkey. Kidney function, free radical contents, and antioxidant enzyme activities were detected by corresponding kits. The expression levels of EndoG, ER stress-related, and apoptosis-related genes were tested by RT-qPCR and Western blotting. The objective is to understand the potential mechanisms underlying AFB1-induced nephrotoxicity in donkeys and provide insights for mitigating AFB1 toxicity.

## 2. Materials and Methods

### 2.1. Animal and Treatment

All procedures were approved by the Animal Welfare and Ethics Committee of the Institute of Animal Science, Liaocheng University (protocol no. 2022112001). Ten 6-month-old weaned male donkey foals (Dezhou donkey, Dezhou, China) with similar weight and body conditions, were randomly and equally divided into two groups, the control group and AFB1-exposed group. The control group was fed a full-price diet (Dong’e Liuhe Lvjia Feed Co., Ltd., Liaocheng, China) and the AFB1-exposed group was fed a full-price diet containing 1 mg AFB1/kg of diet (AFB1, purity ≥ 99.9%, Qingdao Prebon Bioengineering Co. Ltd., Qingdao, China). Donkeys were feed a 1 kg diet at 8:00 a.m. and 15:00 p.m., respectively. Moreover, all foals were housed in individual pens in a donkey barn and had free access to forage grass and water during the experiment. After 30 days of feeding, the foals were slaughtered and blood and kidney samples were collected, processed, and stored in accordance with the study requirements.

### 2.2. Routine Blood and Renal Function Tests

The blood was collected using an EDTA anticoagulant tube and tested in a veterinary automatic blood cell analyzer (BC-5000Vet, Shenzhen Myriad Animal Medical Technology Co., Ltd., Shenzhen, China) to observe the changes in the number and morphological distribution of blood cells.

The serum was obtained from whole blood through centrifugation. It was then added to the BS-180, a fully automatic biochemical analyzer manufactured by Shenzhen Myriad Animal Medical Technology Co., Ltd., China. The analyzer was equipped with test kits specifically designed for measuring the levels of uric acid (UA, D007-a), urea (UREA, D009-a), and creatinine (CREA, D008-a) in the blood.

### 2.3. Determination of Renal Organ Coefficient

The foal’s body weight and the weight of its fresh kidney tissue were measured to calculate the renal organ coefficient of the foal. The formula is as follows:

Renal organ coefficient = kidney weight (kg)/body weight (kg).

### 2.4. Histomorphological Observation

Kidney tissues (1 cm × 1 cm × 0.2 cm) were promptly collected and fixated in a 4% paraformaldehyde buffer (Servicebio, Wuhan Service Biotechnology Co., Wuhan, China) for a minimum of 24 h. The samples underwent the conventional paraffin embedding technique, involving obtaining sections from prepared paraffin blocks. These sections were then degreased with xylene (Chinese national medicine, Beijing Sinopharm Chemical Reagent Co., Ltd., Beijing, China), rehydrated with graded alcohol, and stained with hematoxylin and eosin (Servicebio, Wuhan Service Biotechnology Co., China). The resulting HE-stained sections were examined under a microscope, and images were captured for further analysis.

### 2.5. Ultrastructure Observations

Kidney samples (1 mm^3^) were fixed in a solution of 2.5% glutaraldehyde phosphate sodium buffer (Chinese national medicine, Beijing Sinopharm Chemical Reagent Co., Ltd., China) at a temperature of 4 °C. They were then washed with 0.1 M PBS (pH 7.2, Servicebio, Wuhan Service Biotechnology Co., China) and subsequently treated with osmium tetroxide buffer (Chinese national medicine, Beijing Sinopharm Chemical Reagent Co., Ltd., China) for secondary fixation. To prepare ultrathin sections, a gradient elution using ethanol and acetone (Chinese national medicine, Beijing Sinopharm Chemical Reagent Co., Ltd., China) was performed, followed by soaking and embedding with epoxy. The sections were stained with Mg–uranyl acetate and lead citrate (Chinese national medicine, Beijing Sinopharm Chemical Reagent Co., Ltd., China) and finally examined and imaged using a transmission electron microscope (H-7650, Hitachi Limited, Tokyo, Japan).

### 2.6. TUNEL Assay

Apoptotic cells in kidneys were detected according to the instructions of TUNEL Apoptosis Detection Kit (Servicebio, Wuhan Service Biotechnology Co., China) using the paraffin section. The scanning pictures were observed and obtained using the imaging system of a fluorescence microscope (Nikon DS-U3, Nippon Kogaku Kogyo Co., Tokyo, Japan). DAPI-stained nuclei were blue under UV excitation, and the nuclei of positive apoptotic cells were green.

### 2.7. Immunofluorescence Staining

Paraffin sections of kidney were subjected to antigen retrieval after deparaffinization and rehydration. Then, the sections were blocked with 5% BSA–TBSTx (Solarbio, Beijing Suolaibao Techbology Co., Ltd., Beijing, China) for 1 h at room temperature and incubated with the EndoG (1:200) primary antibody at 4 °C overnight. After washing with TBSTx (Solarbio, Beijing Suolaibao Techbology Co., Ltd., China), sections were incubated with TRITC goat anti-rabbit immunoglobulin G (IgG) for 1 h at room temperature. Finally, DAPI (Solarbio, Beijing Suolaibao Techbology Co., Ltd., China) was used to label the nucleus after washing again. The results were observed and pictures were taken using a fluorescence microscope (IX50; Tokyo Olympus Corporation, Tokyo, Japan), which were analyzed by ImageJ, 1.54k.

To test whether EndoG transferred to the ER, EndoG and GRP78 (ER marker, 1:200) were incubated at one section using homologous double-labeled immunostaining. Other processes were consistent with the general immunofluorescence.

### 2.8. Detection of Antioxidant Function

The contents of total protein (TP, A045-2-2), inducible nitric oxide synthase (iNOS, A014-1), nitric oxide (NO, A012-1), hydrogen peroxide (H_2_O_2_, A064-1-1), malondialdehyde (MDA, A003-1), and the activity of total antioxidant capacity (T-AOC, A015-1) in donkey kidney were detected by spectrophotometry using the supernatant of the kidney homogenate according to the instruction manual of diagnostic kits (Jiancheng Bioengineering Institute, Nanjing, China). The results were calculated with the formula offered in the instructions based on the OD values at 595 nm (TP), 530 nm (iNOS), 550 nm (NO), 405 nm (H_2_O_2_), 532 nm (MDA), and 520 nm (T-AOC).

### 2.9. Real-Time Quantitative PCR Analysis

Total RNA was extracted from kidneys using the classical Trizol method, then total RNA was reverse-transcribed into cDNA using the PrimeScript™ RT reagent Kit with gDNA Eraser (Takara, Dalian Baoji-Medical Technology Co., Ltd., Dalian, China). The cycle threshold (Ct) values of target genes were detected on the CFX96 Touch Real-Time PCR Detection System (Bio-Rad, Bio-Rad Laboratories, Hercules, CA, USA) using TB Green^®®^ Premix Ex Taq™ II (Tli RNaseH Plus) (Takara, Dalian Baoji-Medical Technology Co., Ltd., Kusatsu City, Japan). The reaction system was as follows: TB Green^®®^ Premix Ex Taq™ II, 12.5 μL; forward primer, 1 μL; reverse primer, 1 μL; cDNA, 2 μL; and DEPC H_2_O, 8.5 μL. Amplification procedures were as follows: 95 °C for 30 s; 95 °C for 5 s; and 60 °C for 30 s, 39 cycles. Table 1 lists the primers used in this study, and the primer solubilization curves are single peaks. The relative mRNA expression levels of target genes were calculated by the method of 2^−ΔΔCt^ based on the internal reference gene actin (β-actin).

### 2.10. Western Blotting Analysis

Total proteins were extracted from kidneys by radioimmunoprecipitation assay (RIPA) lysis buffer (Beyotime, Shanghai Biyuntian Biotechnology Co., Ltd., Shanghai, China) with the addition of 1 mM PMSF (Beyotime, Shanghai Biyuntian Biotechnology Co., Ltd., China) and 2% phosphatase inhibitor (Beyotime, Shanghai Biyuntian Biotechnology Co., Ltd., China). The protein concentration was then determined according to the instructions of BCA Protein Concentration Assay Kit (Solarbio, Beijing Solarbio Technology Co., Ltd., China). Equal amounts of total protein were subjected to SDS-PAGE gel electrophoresis and transferred onto a nitrocellulose membrane (Biosharp, Beijing Lanjieke Science and Technology Co., Ltd., Beijing, China). The membranes were blocked in 5% TBST–skim milk for 1 h at 37 °C and then incubated with the primary antibody at 4 °C overnight. After washing three times, the membranes were incubated with HRP goat anti-rabbit IgG (1:10,000, Abclonal, Wuhan ABclonal Technology Co., Ltd., Wuhan, China) at room temperature for 120 min. The positive signal was detected on the chemiluminescence system using an enhanced chemiluminescence kit (Meilunbio, Dalian Meilun Biotechnology Co., Ltd., Dalian, China). Images were collected by the ChemiDocTM XRS+ imaging system and then analyzed by Image J. β-actin was used as an internal reference. The primary antibodies used in this study are listed in Table 2.

### 2.11. Data and Analyses

The experimental data were statistically analyzed using WPS office and graphically analyzed using GraphPad Prism 8.0.2. Data are expressed as mean ± SEM (*n* = 5), and the differences between the two groups were analyzed using the *t*-test. “*” denotes a significant difference compared to the control group (*p* < 0.05); “ns” indicates no significant difference compared to the control group (*p* > 0.05).

## 3. Results

### 3.1. Effect of AFB1 on Blood Indices in Donkey

As shown in Table 3, compared to the control group, the AFB1 group did not show any significant effects on white blood cell counts (WBC), hemoglobin (HGB), platelets (PLTs), red blood cell counts (RBC), and lymphocytes (Lym). However, there was a significant increase in neutrophil number (Neu), neutrophil percentage (Neu %), and lymphocyte percentage (Lym %) in the AFB1 group (*p* < 0.05). These findings suggest that AFB1 exposure leads to inflammation in donkeys, disrupting their homeostasis and causing damage.

### 3.2. Impact of AFB1 on Renal Organ Coefficient and Renal Function in Donkeys

**Figure 2:**
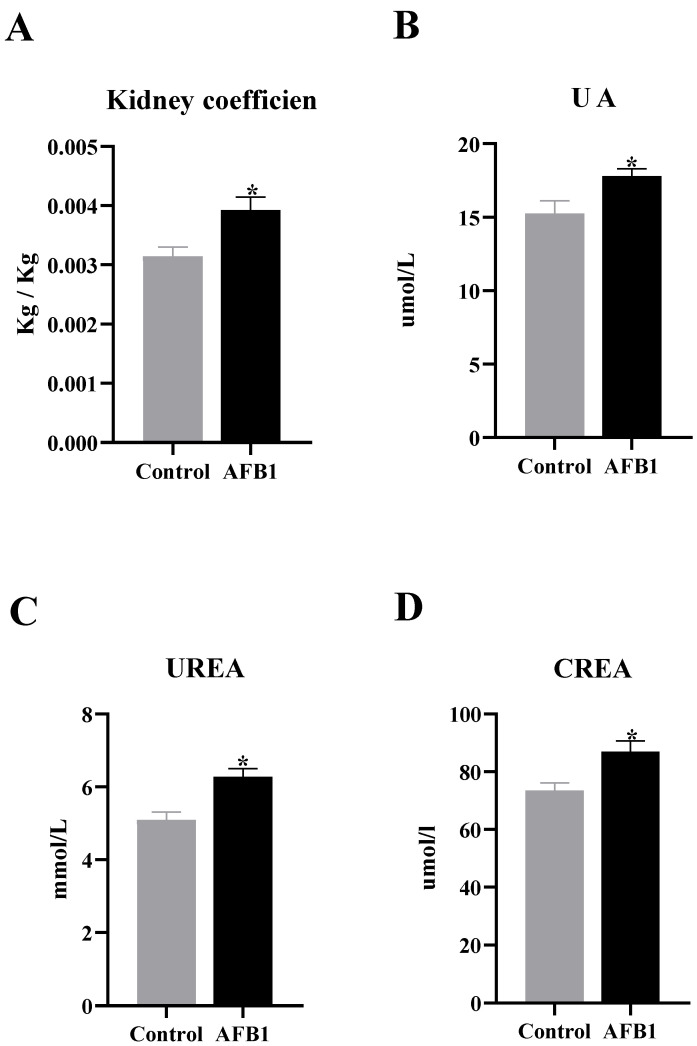
Determination of renal organ coefficients and renal function tests in donkeys. (**A**) Kidney organ coefficient; (**B**) UA; (**C**) UREA; (**D**) CREA, “*” denotes a significant difference compared to the control group (*p* < 0.05).

As shown in Figure 2A, the renal organ coefficient in the AFB1 group was significantly higher compared to the control group (*p* < 0.05). Additionally, the levels of UA, CREA, and UREA in the serum were significantly elevated in the AFB1 group (*p* < 0.05) (Figure 2B–D). These findings suggest that AFB1 exposure could result in kidney enlargement and impair kidney function in donkeys.

### 3.3. Effect of AFB1 on Histopathology of Donkey Kidney Tissue

**Figure 3:**
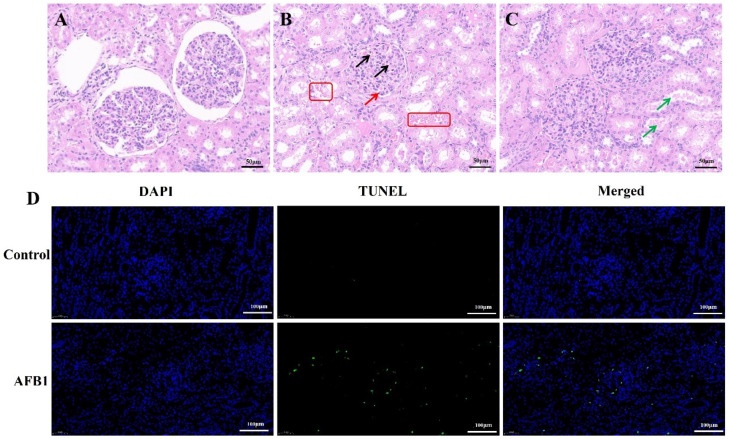
Kidney histopathological observation in donkey. (**A**–**C**) HE staining results ((**A**) control group; (**B**,**C**) AFB1 group); (**D**) TUNEL staining results. The original images in the manuscript are published as Supplementary Materials.

The kidney tissues in the control group displayed normal morphologies. The glomerulus and its surrounding renal tubules were intact and not detached. The lumen of the balloon was clearly visible (Figure 3A). However, the kidney tubular epithelial cells were congested and detached in the AFB1 group (Figure 3B, red frame). The glomerulus was also congested (Figure 3B, black arrows), and there was a reduction in the gap between the glomerulus and renal capsule (Figure 3B, red arrow). Additionally, casts appeared in the renal tubules (Figure 3C, green arrow). Furthermore, the TUNEL staining results shown that AFB1 exposure increased the number of apoptotic cells compared to the control group (Figure 3D). These findings demonstrate that AFB1 caused pathological damage and apoptosis in the donkey kidney. 

### 3.4. Effect of AFB1 on Ultrastructure of Kidney in Donkey

**Figure 4:**
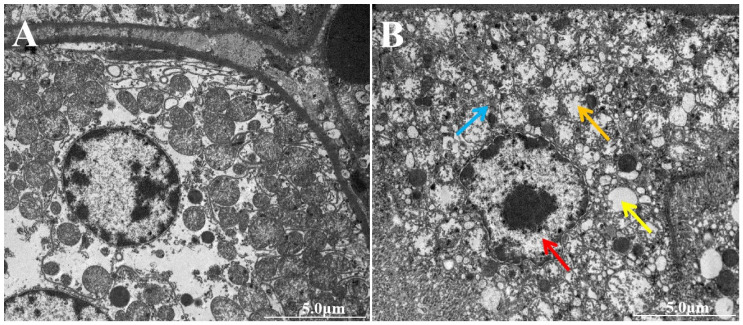
The ultrastructure observation of kidney in donkey. (**A**) Control group; (**B**) AFB1 group.

As shown in Figure 4A, the control group did not exhibit any obvious abnormalities. However, the AFB1 group, when compared to the control group, displayed typical apoptotic features in the ultrastructure of the kidney. These features included nuclear wrinkling, nuclear deformation, and chromatin aggregation (Figure 4B, red arrow). Additionally, there was evidence of mitochondrial aggregation, swelling, disappearance of crests, and vacuolization (Figure 4B, orange arrows), as well as the accumulation of lipid droplets (Figure 4B, yellow arrows) and the dissolution and fragmentation of the ER (Figure 4B, blue arrows). These findings suggest that exposure to AFB1 can induce apoptosis in the kidney, with ER and mitochondrial damage likely playing significant roles.

### 3.5. Effect of AFB1 on Antioxidant Capacity in Kidney of Donkey

**Figure 5:**
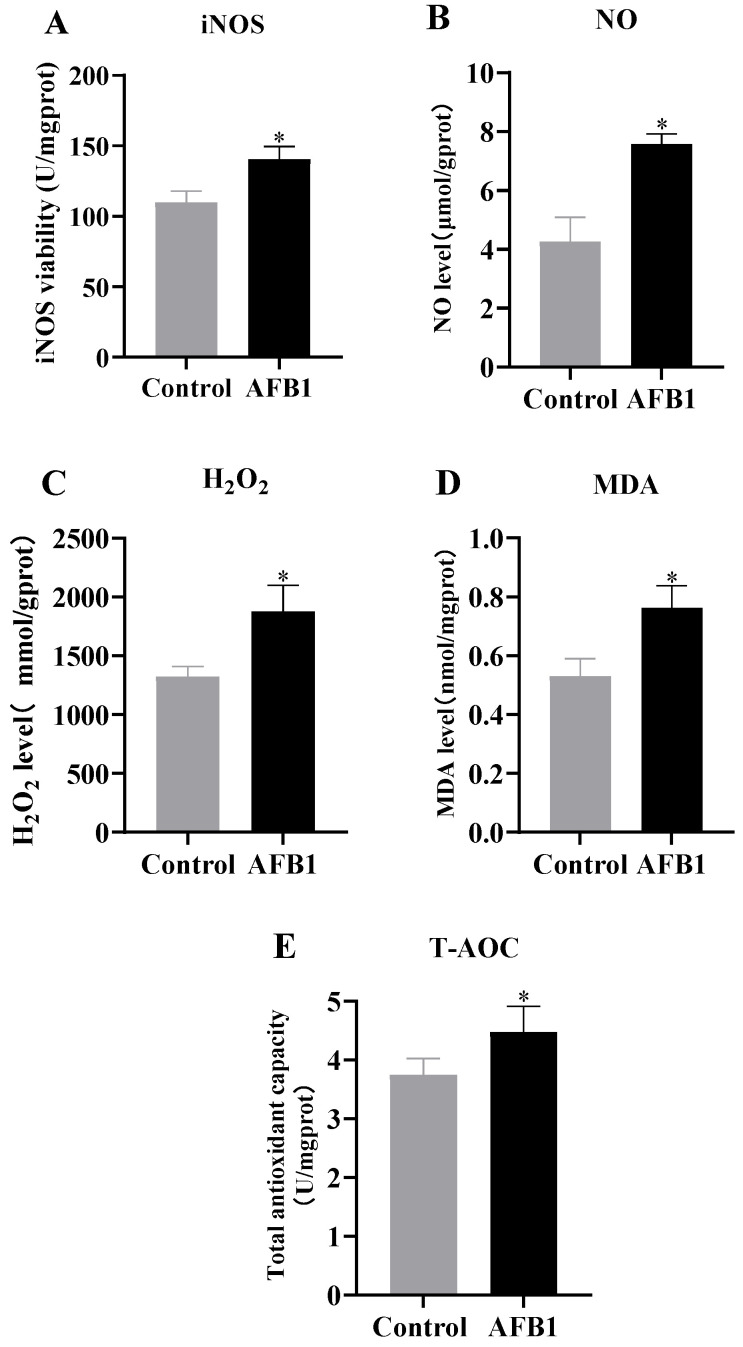
Levels of antioxidant capacity of donkey kidney. (**A**) iNOS; (**B**) NO; (**C**) H_2_O_2_; (**D**) MDA; (**E**) T-AOC, “*” denotes a significant difference compared to the control group (*p* < 0.05).

As shown in Figure 5, the AFB1-exposure-group donkeys showed significantly higher levels of iNOS (Figure 5A), NO (Figure 5B), H_2_O_2_ (Figure 5C), MDA (Figure 5D), and T-AOC (Figure 5E) in kidney compared with the control group (*p* < 0.05). The results indicate that AFB1 exposure causes oxidative stress to the kidneys of donkeys and a compensatory increase in antioxidant capacity to cope with oxidative stress.

### 3.6. Effect of AFB1 on the EndoG Expression in Donkey Kidney

**Figure 6:**
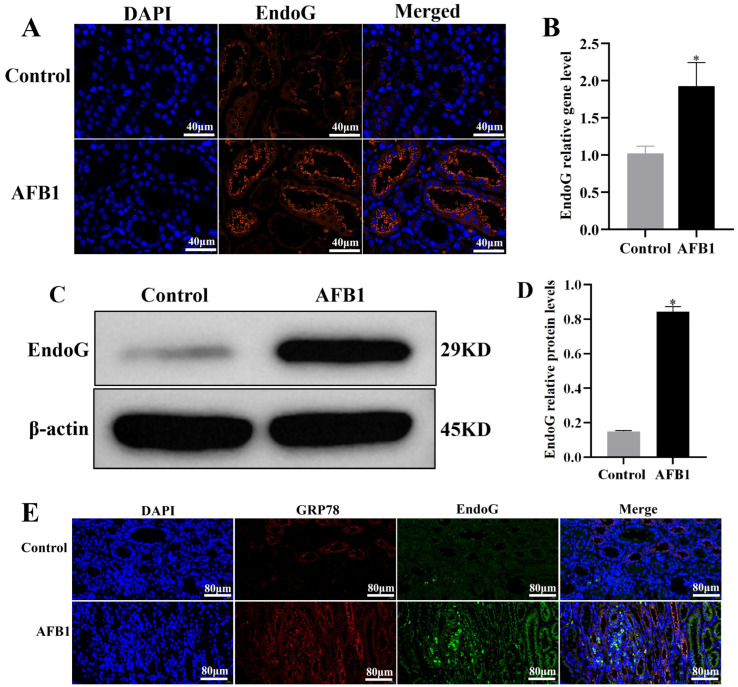
EndoG expression detection in donkey kidney. (**A**) EndoG immunofluorescence results; (**B**) mRNA expression; (**C**) Western blot results; (**D**) protein expression; (**E**) co-immunofluorescence staining of GRP78 and EndoG, where red represents GRP78, green represents EndoG, and blue represents nuclei, “*” denotes a significant difference compared to the control group (*p* < 0.05).

As shown in Figure 6, the immunofluorescence staining (Figure 6A), mRNA expression (Figure 6B), and protein expression (Figure 6C) results of EndoG confirmed that AFB1 exposure increased the expression of EndoG (*p* < 0.05). Moreover, the immunofluorescence double-staining results of EndoG and GRP78 show that AFB1 exposure increases the expression of GRP78 and the colocalization of EndoG and GRP78 (ER) (Figure 6E). These results suggest that AFB1 exposure induces EndoG expression and promotes EndoG translocation to the ER and binds to GRP78 in donkey kidneys.

### 3.7. Effects of AFB1 Exposure on ER Stress in Donkey Kidney

**Figure 7:**
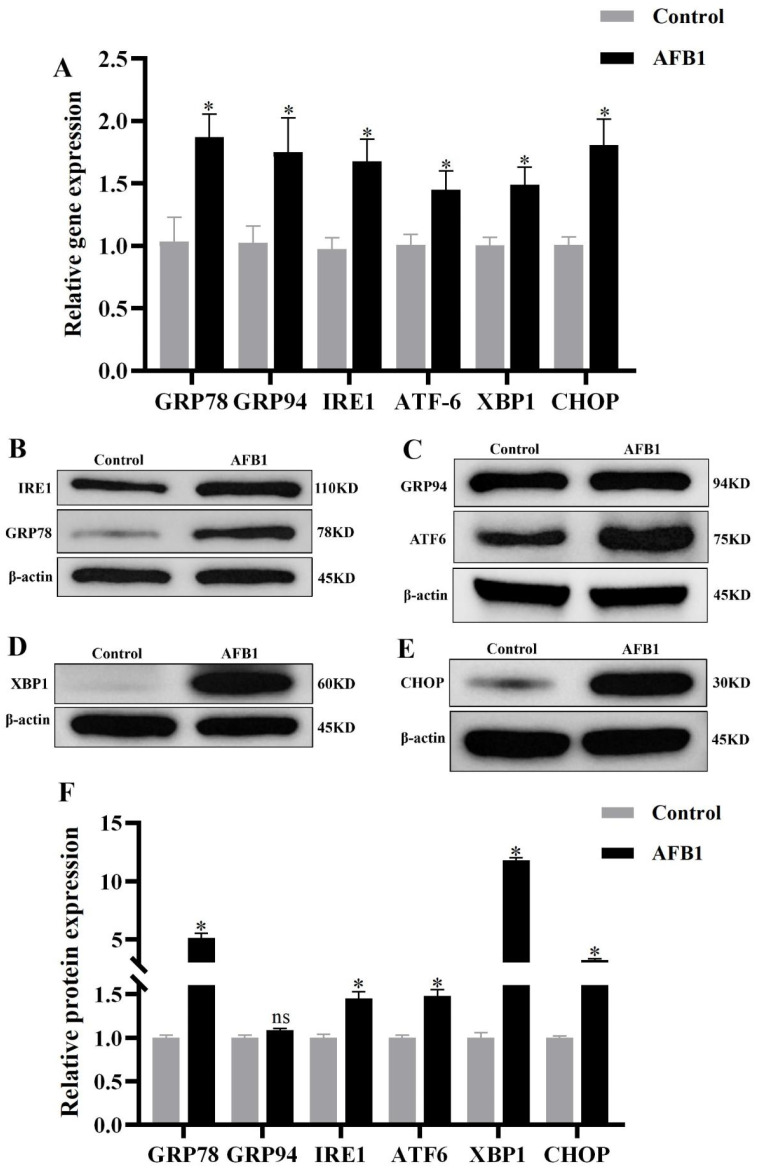
ER stress-related mRNA and protein expression. (**A**) mRNA expression; (**B**–**E**) Western blot results; (**F**) protein expression, “*” denotes a significant difference compared to the control group (*p* < 0.05); “ns” in-dicates no significant difference compared to the control group (*p* > 0.05).

As shown in Figure 7, the ER stress marker GRP78 was significantly elevated at both the gene and protein levels (*p* < 0.05, Figure 7A,B,F). GRP94 was significantly expressed at the gene level (*p* < 0.05, Figure 7A) but not at the protein level (*p* > 0.05, Figure 7C,F). The expressions of ATF-6, IRE1, XBP1, and CHOP were significantly elevated at gene and protein levels (*p* < 0.05, Figure 7A–F). These results indicate that AFB1 exposure induces ER stress in the donkey kidney, and it might promote the expression of CHOP through activating the IRE1/ATF6 pathway.

### 3.8. Effects of AFB1 Exposure on Apoptosis in Donkey Kidney

**Figure 8:**
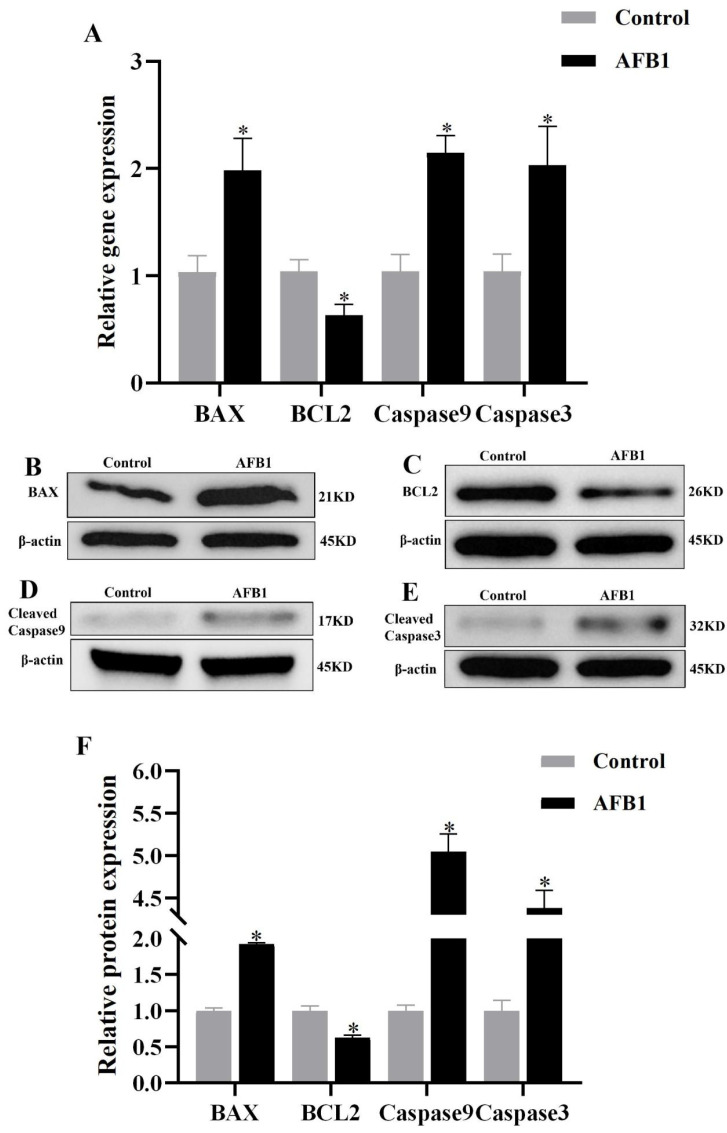
Apoptosis-related mRNA and protein expression. (**A**) mRNA expression; (**B**–**E**) Western blot results; (**F**) protein expression, “*” denotes a significant difference compared to the control group (*p* < 0.05).

As shown in Figure 8, the mRNA and protein expression levels of BAX (Figure 8A,B,F), caspase9 (Figure 8A,D,F), and caspase3 (Figure 8A,E,F) in the AFB1 group were significantly higher than the control group (*p* < 0.05). Conversely, BCL2 exhibited a significantly lower expression (*p* < 0.05, Figure 8A,C,F). These results indicated that the AFB1 exposure activated the mitochondrial apoptotic pathways.

## 4. Discussion

AFB1 is one of common contaminants in animal feed and forage grasses; AFB1 exposure could cause damage to animal organisms such as hepatotoxicity, nephrotoxicity, and neurotoxicity, and it poses a potential health risk to humans [25,26,27,28,29,30,31]. In this study, we first demonstrated the partial mechanism by which AFB1 causes kidney injury in donkey. These data from the AFB1-exposure donkey foal model confirmed that prolonged exposure to AFB1 led to kidney damage in donkey, including swelling and hemorrhage, subcellular organelle injury, and oxidative stress. Furthermore, AFB1 exposure caused a high expression of EndoG, translocation to the ER, and binding to GRP78, and it also induced ER stress (ATF6 and IRE1 pathway), which eventually activated the mitochondrial apoptosis pathway.

It is widely acknowledged that AFB1 exposure affects various animals and humans in terms of health condition and life safety. AFB1 exposure can impair digestion in pigs, break down the intestinal barrier, and inhibit their growth [11]. Cattle exposed to AFB1 exhibit behavioral changes, such as depression and anorexia, and organ changes such as liver enlargement, gallbladder enlargement, and congestion of the intestines and kidneys [12]. A routine blood test is a very convenient and fast inspection, and it is also necessary for basic inspections to determine whether the body is in an abnormal condition. It was found that Neu, Neu %, and Lym % were increased by AFB1 exposure, which indicated that AFB1 exposure caused inflammation in donkeys, disrupted their homeostasis, and induced damage. This result was similar to the research of Oa Mogilnaya et al. in mice in contrast to the study by Raúl Bodas et al. in cows [32,33]. This difference may be attributed to species differences or the duration of AFB1 exposure. Kidney injury was considered as one characteristic of AFB1 exposure. UA, CREA, and UREA are important indexes for kidney function, whose contents commonly can increase under kidney impairment caused by various toxic substance exposures. Chromium or zearalenone exposure causes kidney damage and elevated UA and CREA in mice [34,35]. AFB1 exposure similarly resulted in a significant elevation of UA and CREA in mice and chickens [36,37]. In this study, the contents of UA, CREA, and UREA were increased in AFB1-exposed donkeys, which suggested that AFB1 exposure could cause kidney damage in donkey as well as the animals mentioned above.

Oxidative stress is the key mechanism of AFB1 toxicity. AFB1 disturbs the activities of antioxidant enzymes, leading to peroxide accumulation and excessive ROS-induced damage to proteins and nucleic acids, resulting in the production of large amounts of MDA. AFB1 exposure induces oxidative stress by causing a significant elevation of MDA and T-AOC in the testes of sheep [38]. AFB1 exposure was able to cause a significant elevation of H_2_O_2_, MDA, and T-AOC in the kidney tissues of mice and a significant elevation of MDA and T-AOC in the kidney tissues of rabbits, causing oxidative stress [39,40]. We found the iNOS activity, H_2_O_2_, NO, and MDA contents were increased in AFB1-exposure donkey kidneys, which indicated that AFB1 exposure induced oxidative stress, causing kidney oxidative damage. Moreover, the T-AOC was also increased in AFB1-exposure donkey kidneys, which may be a compensatory increase against oxidative damage. Oxidative stress can cause damage to organelles, especially mitochondria [41]. As the centers of intracellular redox reactions, mitochondria are the main targets of oxidative stress, and long-term oxidative stress may lead to mitochondrial damage and dysfunction [42,43].

EndoG, an endonuclease found in the inner membrane gap of mitochondria, is released into the cytoplasm when mitochondrial homeostasis is disrupted by an external stimulus [44]. Furthermore, EndoG in the cytoplasm can further enter the ER under conditions of a high-fat diet and ER stress inducers, binding to Bip to elicit an ER stress response [17]. It was shown that GRP78 and GRP94 were significantly elevated in chicken bursa and thymus by AFB1 exposure, and GRP78, IRE1, XBP1, and CHOP were significantly elevated in mouse hippocampal neurons; thus, exposure to AFB1 resulted in ER stress [45,46,47]. In this study, AFB1 exposure upregulated the expression of EndoG and GRP78 and led to EndoG accumulation in the ER, identifying AFB1 exposure-induced ER stress in donkey kidney. ER transmembrane sensors (PERK, IRE1, and ATF6) can relieve ER stress and restore ER homeostasis by reducing the accumulation of ER proteins [48]. However, continuing ER stress triggers the activation of the apoptotic pathway, ultimately resulting in cell apoptosis [49]. CHOP is an important molecule involved in the transition of ER stress from anti-apoptosis to pro-apoptosis [50]. The activation of PERK, XBP1, and ATF6 can mediate the production of CHOP, which promotes apoptosis [51,52]. In the present study, AFB1 exposure activated the IRE1/ATF6-CHOP pathway, which caused endoplasmic reticulum stress in renal tissues.

Mitochondria play a crucial role in cell apoptosis. Mitochondria receive apoptotic signals released by external stimuli, which lead to mitochondrial outer membrane permeability (MOMP) and trigger the release of a series of proteins within the mitochondria, inducing the mitochondrial apoptosis pathway [37]. The BCL2 family plays key roles in regulating mitochondrial outer membrane integrity and function. CHOP plays a crucial role in this process due to its regulation on the BCL2 family of proteins, specifically down-regulating BCL2 expression and increasing the expression of BAX [53,54]. As a pore-forming protein, BAX transfers from the cytoplasm to the mitochondria, leading to mitochondrial outer membrane permeability (MOMP) and triggering apoptosis. The inhibition of BCL2, the transference of BAX to mitochondria, the release of cytochrome C into the cytosol, and the activation of apoptotic initiators and executioners (for caspases) primarily involve several key events of the mitochondrial apoptotic pathway [55,56,57]. Studies have shown that exposure to AFB1 increases BAX and caspase3 levels, decreases BCL2 levels, activates the mitochondrial apoptotic pathway, and leads to apoptosis in mouse testes and chicken liver [58,59]. The Tunel and ultrastructure observation results showed that AFB1 induced donkey kidney cell apoptosis. Additionally, the decrease in BCL2 expression and the increase in BAX, caspase9, and caspase3 expression suggested that AFB1 exposure activated the mitochondrial apoptosis pathway.

## 5. Conclusions

Altogether, our study concluded that the donkey kidney exhibits notable sensitivity to AFB1, resulting in pathological changes. Furthermore, AFB1 exposure promotes the production of ROS, leading to oxidative stress in the donkey kidney. In response, a compensatory increase in T-AOC was observed. Mechanistic investigations further revealed that AFB1 exposure activated EndoG, which triggered ER stress and subsequently activated the IRE1/ATF6-CHOP signaling pathway. This cascade ultimately induced mitochondrial apoptosis. As a result, AFB1 exposure caused significant kidney damage and dysfunction. Overall, our findings contribute to a deeper understanding of the mechanisms underlying AFB1-induced nephrotoxicity in donkeys. Importantly, these results provide a foundation for future research aimed at mitigating AFB1 toxicity, such as strategies to alleviate oxidative stress through ROS removal or the enhancement of antioxidant defenses, as well as approaches to inhibit EndoG expression and alleviate ER stress to reduce kidney injury.

## Figures and Tables

**Table 1:** Primers used in this study.

Name	Forward Primer Sequence (5′-3′)	Reverse Primer Sequence (5′-3′)
β-actin	CGGGACCTGACGGACTACCTC	TCCTTGATGTCACGCACGATTTCC
Endo G	TCTGCTCCTATGTGATGCCCAAC	CTTGACTCTGCCCGCCCTTG
GRP78	AACCGCATCACGCCGTCTTATG	GGTTGGAGGTGAGCTGGTTCTTG
GRP94	ACCCCGATGCAAAGGTTGAAGAAG	GTCTTGCTCCGTGTCGTCTGTG
ATF-6	TGGGAAACAGGCATTTGGGACATC	CTGAACAACTTGAGGAGGCTGGAG
IRE1	TCCAACCACTCGCTCCACTCTAC	CCTCATCCTCGTCGTCCTGCTC
XBP1	ACTGAAGAGGAGGCTGAGACCAAG	GGAGAGGTTCTGGAGGGGTGAC
CHOP	TGCTTCTCTGGCTTGGCTGAC	TGGTCTTCCTCCTCTTCCTCCTG
BAX	TGGACACTGGACTTCCTTCGAGAG	TGGTGAGCGAGGCGGTGAG
BCL2	GGGACGCTTTGCCACGGTAG	CGGTTGACGCTCTCCACACAC
Caspase9	GACCTGACCGCCGAGCAAATG	TGACAGCCGTGAGAGAGGATGAC
Caspase3	TGCAGAAGTCTAGCTGGAAAACCC	TAGCACAAAGCGACTGGATGAACC

**Table 2:** Brands and dilutions of primary antibodies.

Antibody Name	Diluting Factor	Source	Brand
β-actin	1:10,000	Rabbit	Abclonal
EndoG	1:1000	Rabbit	Abclonal
GRP78	1:2000	Rabbit	Wanleibio
GRP94	1:1000	Rabbit	Wanleibio
ATF6	1:2000	Rabbit	Wanleibio
IRE1	1:1000	Rabbit	Wanleibio
XBP1	1:1000	Rabbit	Wanleibio
BAX	1:1000	Rabbit	Wanleibio
BCL2	1:1000	Rabbit	Wanleibio
Caspase9	1:1000	Rabbit	Wanleibio
Caspase3	1:1000	Rabbit	Wanleibio

Note: Abclonal, Wuhan ABclonal Technology Co., Ltd., China; Wanleibio, Shenyang Wanbei Biotechnology Co., Shenyang, China.

**Table 3:** Routine testing of donkey blood.

	Control Group	AFB1 Group	*p*-Value
WBC (10^9^/L)	14.73 ± 1.23	13.94 ± 0.88	0.27
HGB (g/L)	117.2 ± 9.83	105.6 ± 10.6	0.11
PLT (10^9^/L)	301 ± 72.6	328.25 ± 36.3	0.53
RBC (10^12^/L)	6.88 ± 0.51	6.22 ± 0.72	0.133
Lym (10^9^/L)	6.82 ± 1.85	7.8 ± 1.92	0.194
Neu (10^9^/L)	4.91 ± 0.29	6.52 ± 0.78	0.002
Neu%	35.3 ± 3.17	44.62 ± 7.38	0.03
Lym%	46.14 ± 6.14	55.82 ± 3.67	0.015

Note: Values for control and AFB1 are mean ± standard deviation, and *p*-values were derived from *t*-tests.

## Data Availability

All the data are available in this article.

## Supplementary Materials

The following are available online at https://www.mdpi.com/article/10.3390/vetsci12020130/s1, The original images in the manuscript are published in the Supplementary Materials.
