# Supplementary figures and images for: Aflatoxin B1-Induced Apoptosis in Donkey Kidney via EndoG-Mediated Endoplasmic Reticulum Stress
Source: Vet Sci. 2025 Feb 5;12(2):130. doi: 10.3390/vetsci12020130 (PMC11860441; doi:10.3390/vetsci12020130)

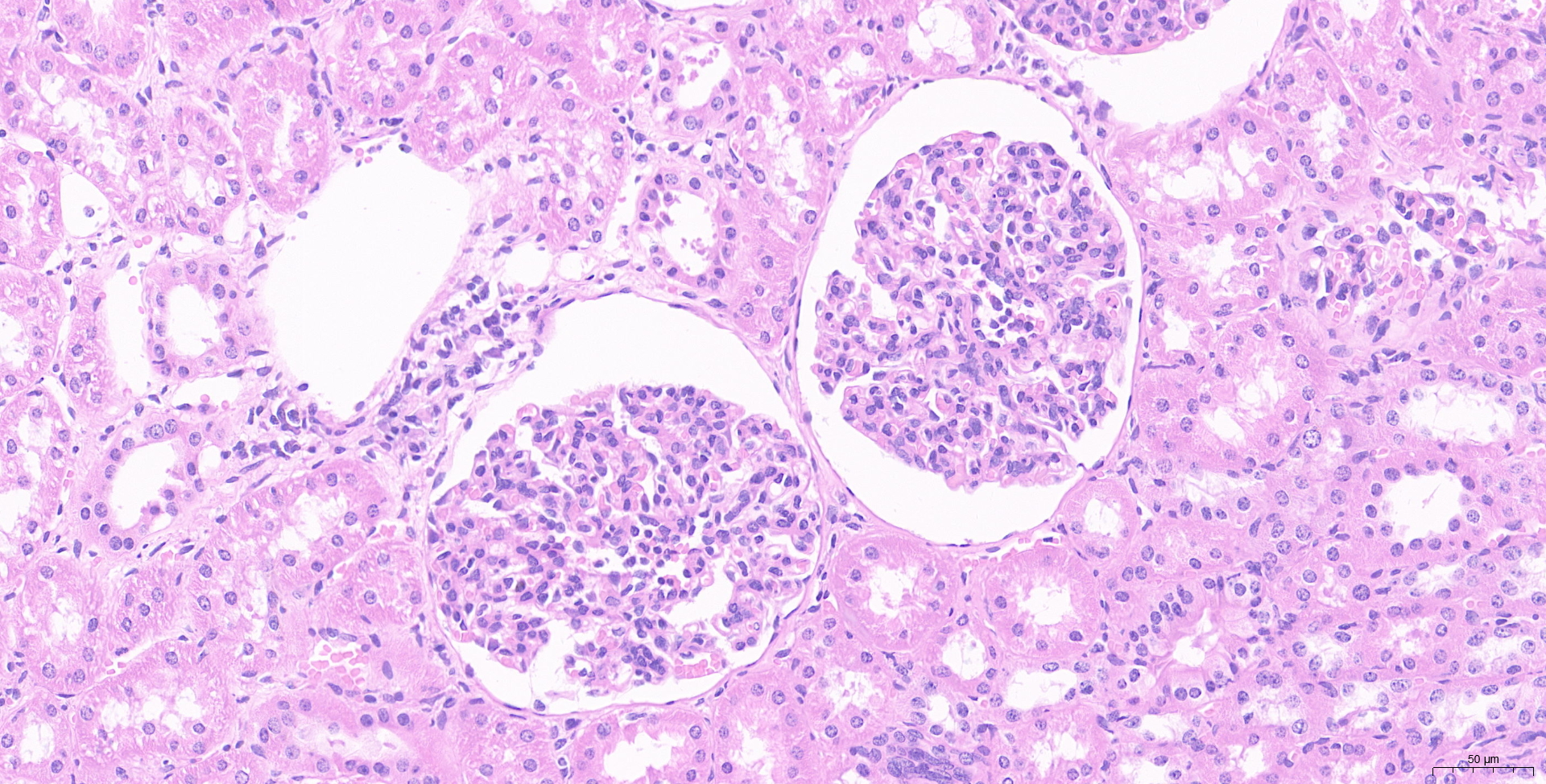

Supplement: Supplementary file 1 [file vetsci-12-00130-s001.zip › TEM、HE and IHC/Fig3-A.jpg]

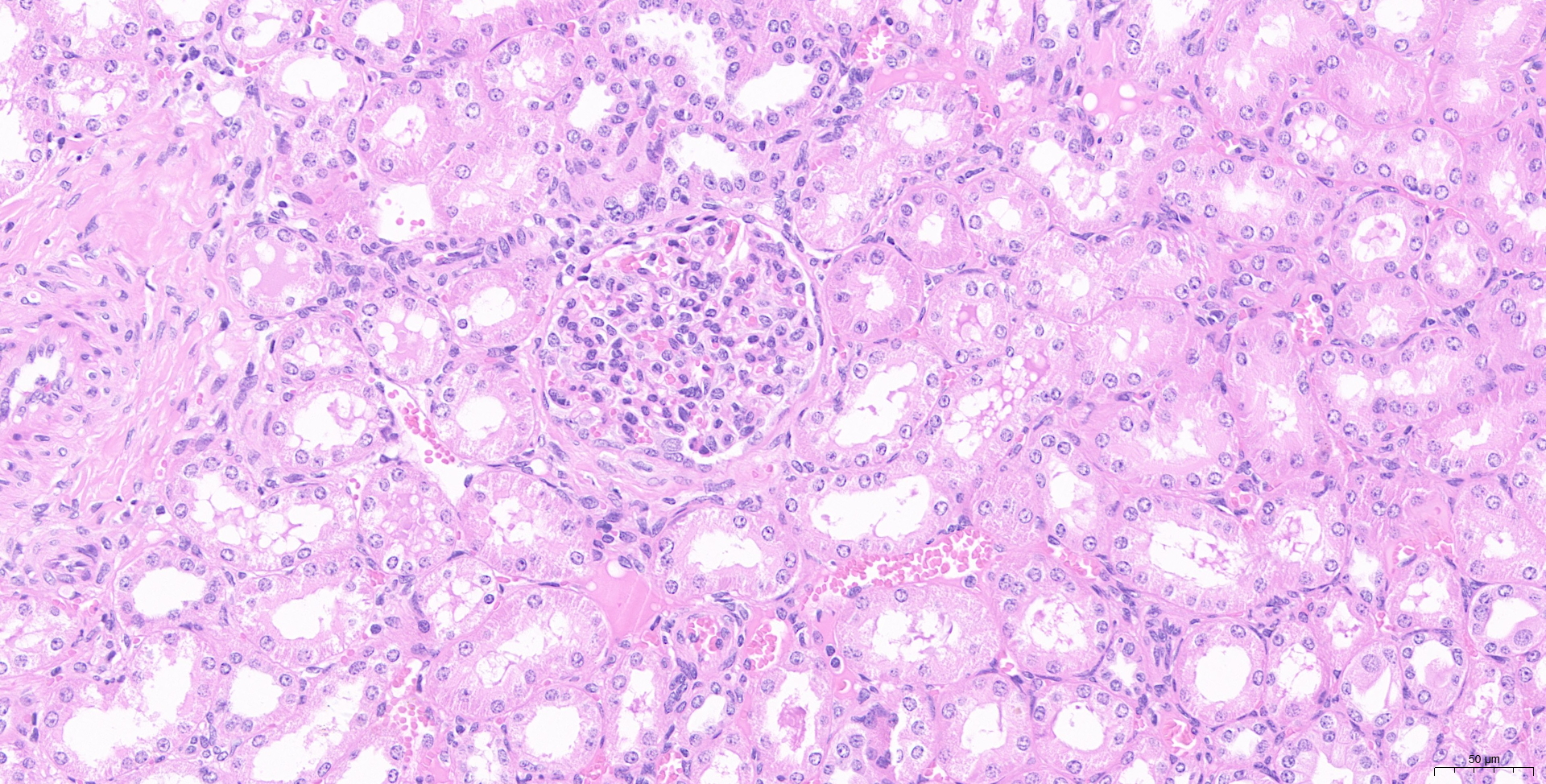

Supplement: Supplementary file 1 [file vetsci-12-00130-s001.zip › TEM、HE and IHC/Fig3-B.jpg]

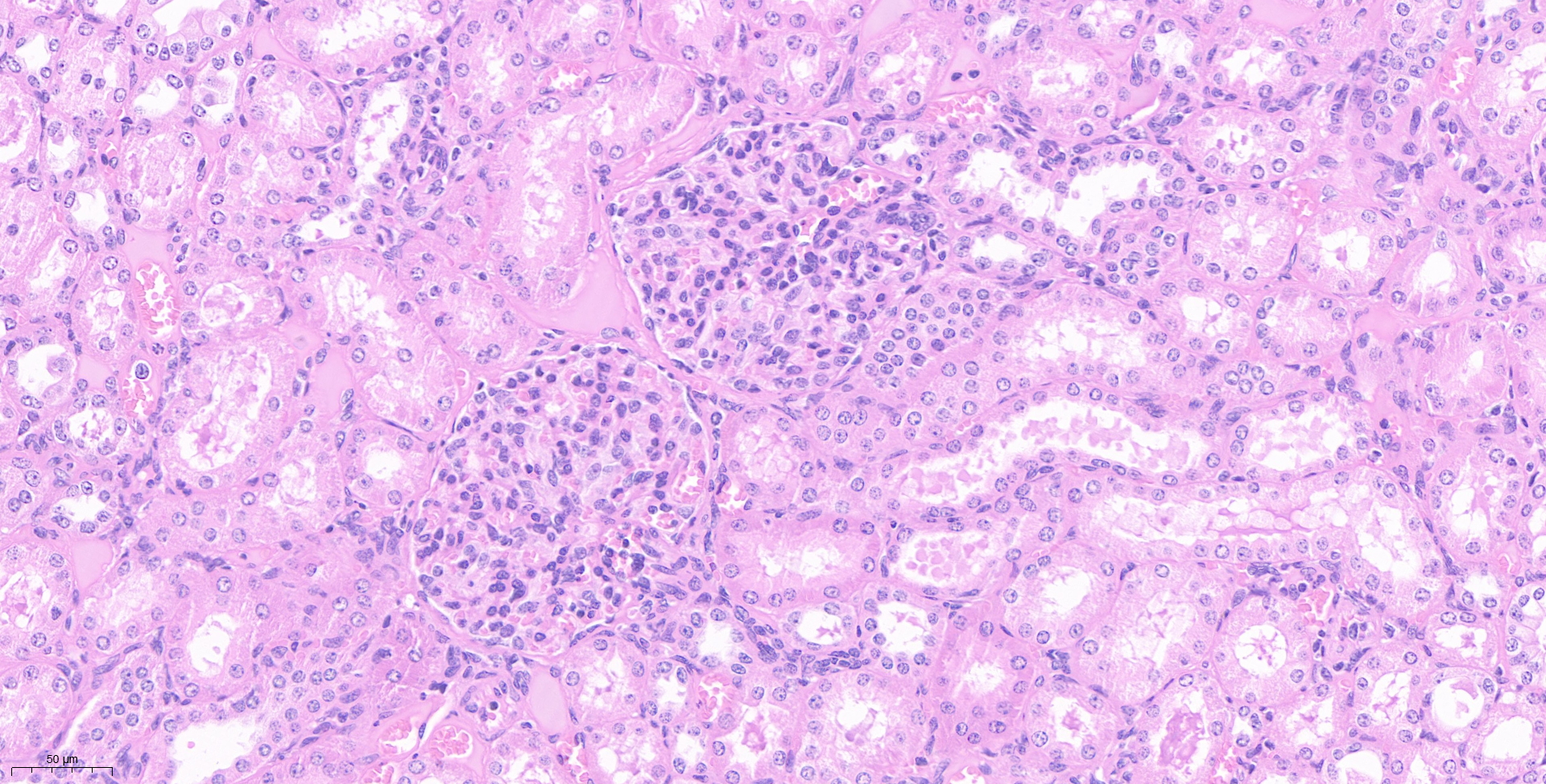

Supplement: Supplementary file 1 [file vetsci-12-00130-s001.zip › TEM、HE and IHC/Fig3-C.jpg]

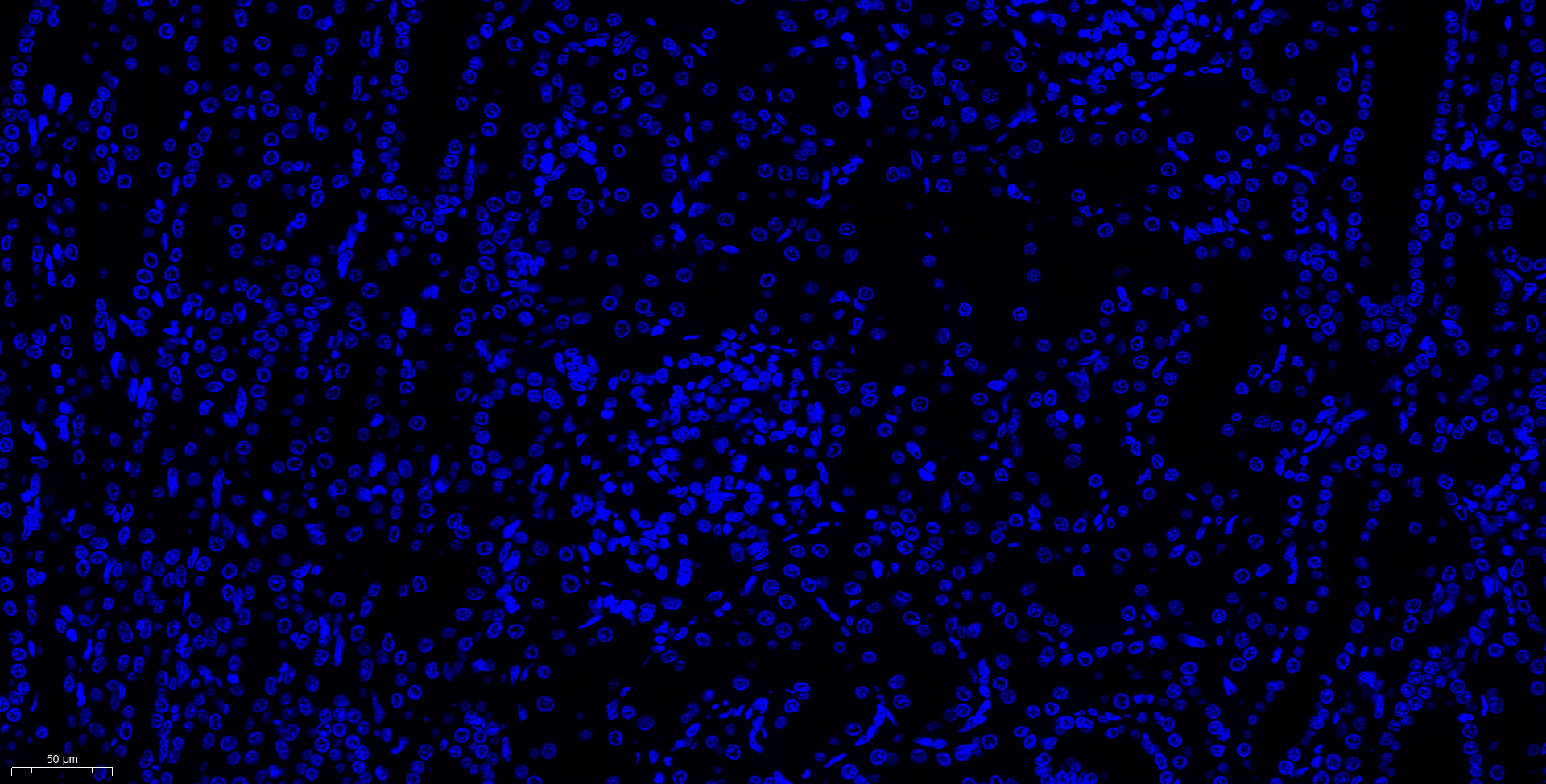

Supplement: Supplementary file 1 [file vetsci-12-00130-s001.zip › TEM、HE and IHC/Fig3-D1.jpg]

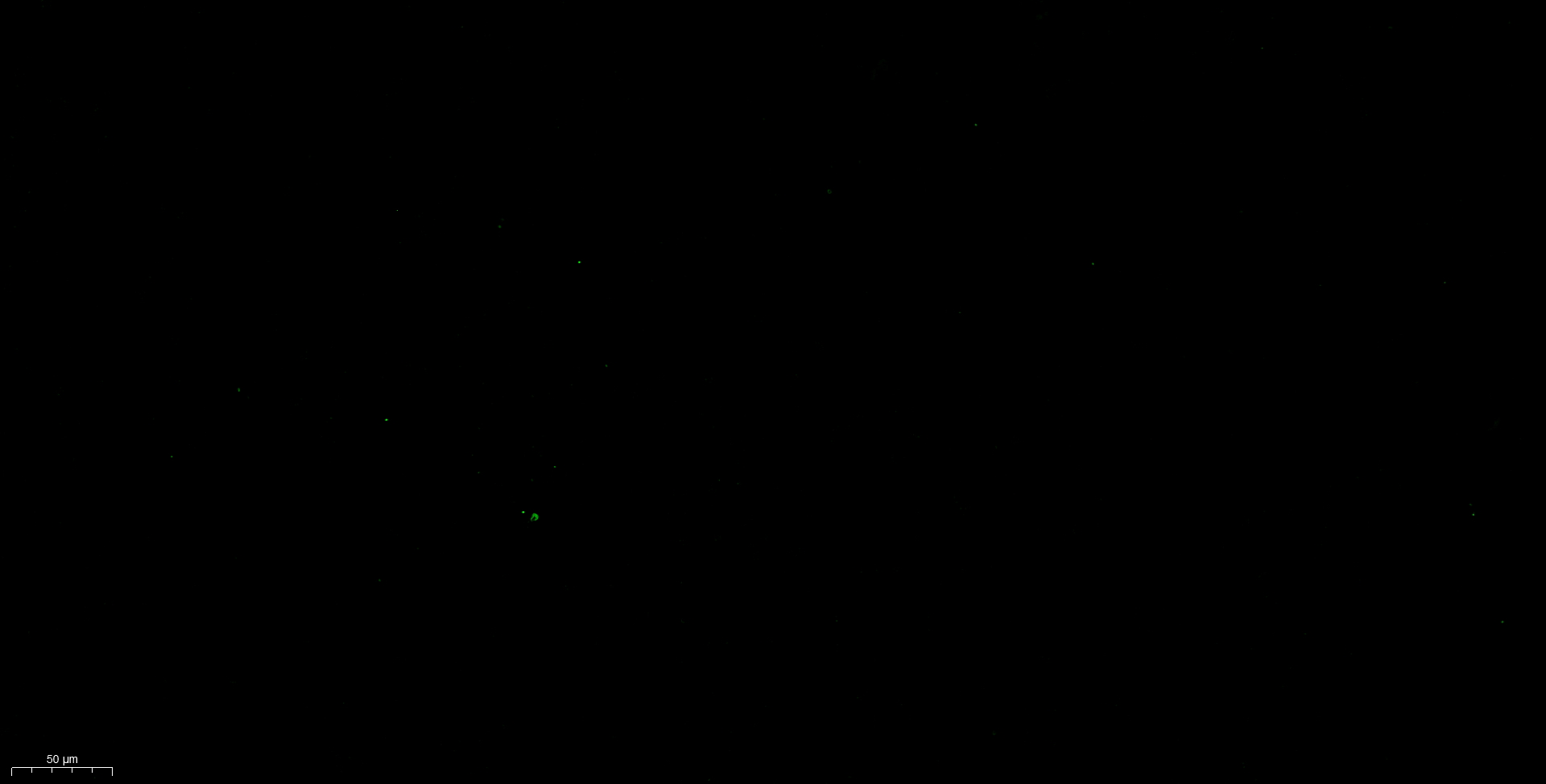

Supplement: Supplementary file 1 [file vetsci-12-00130-s001.zip › TEM、HE and IHC/Fig3-D2.jpg]

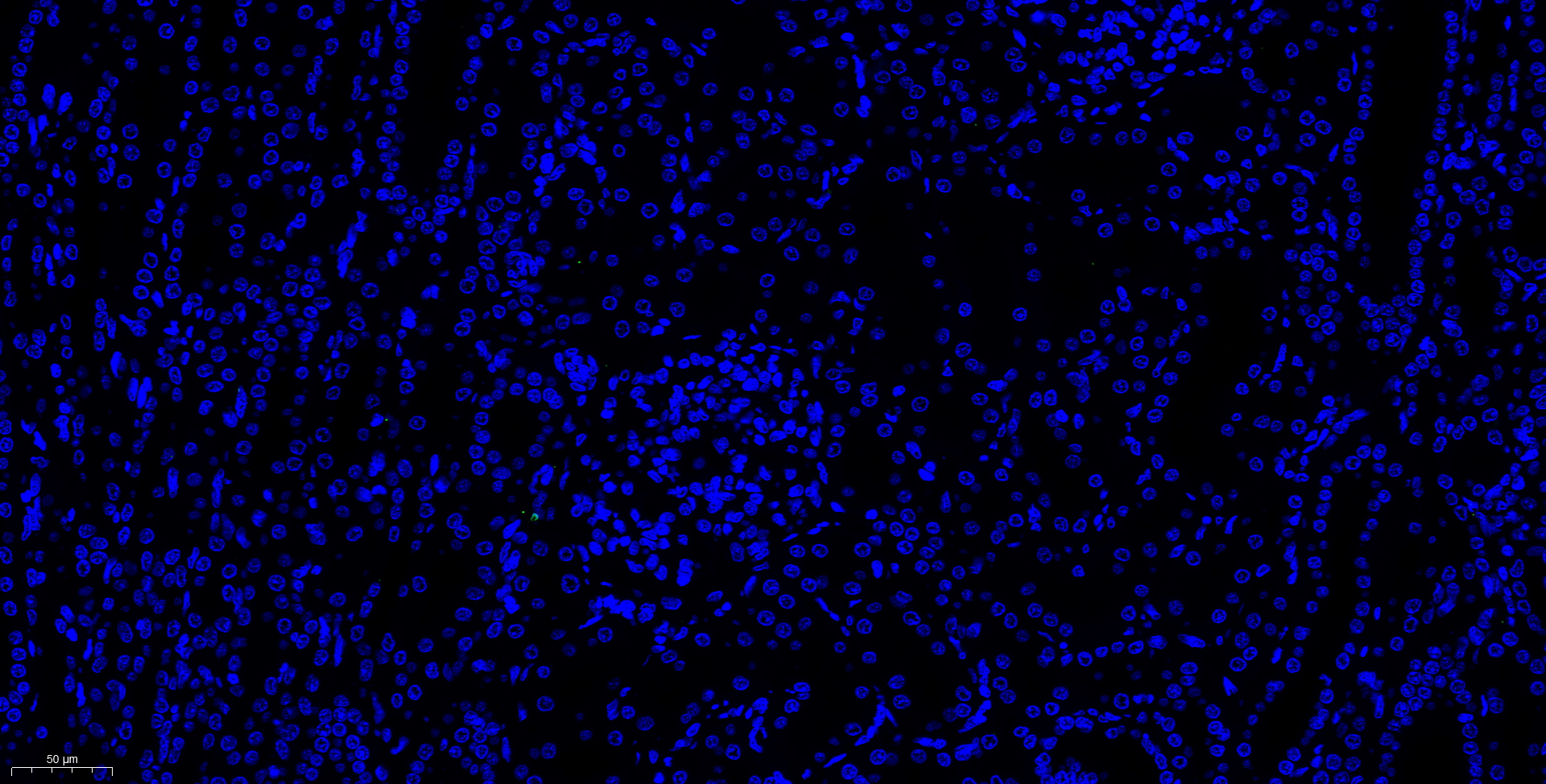

Supplement: Supplementary file 1 [file vetsci-12-00130-s001.zip › TEM、HE and IHC/Fig3-D3.jpg]

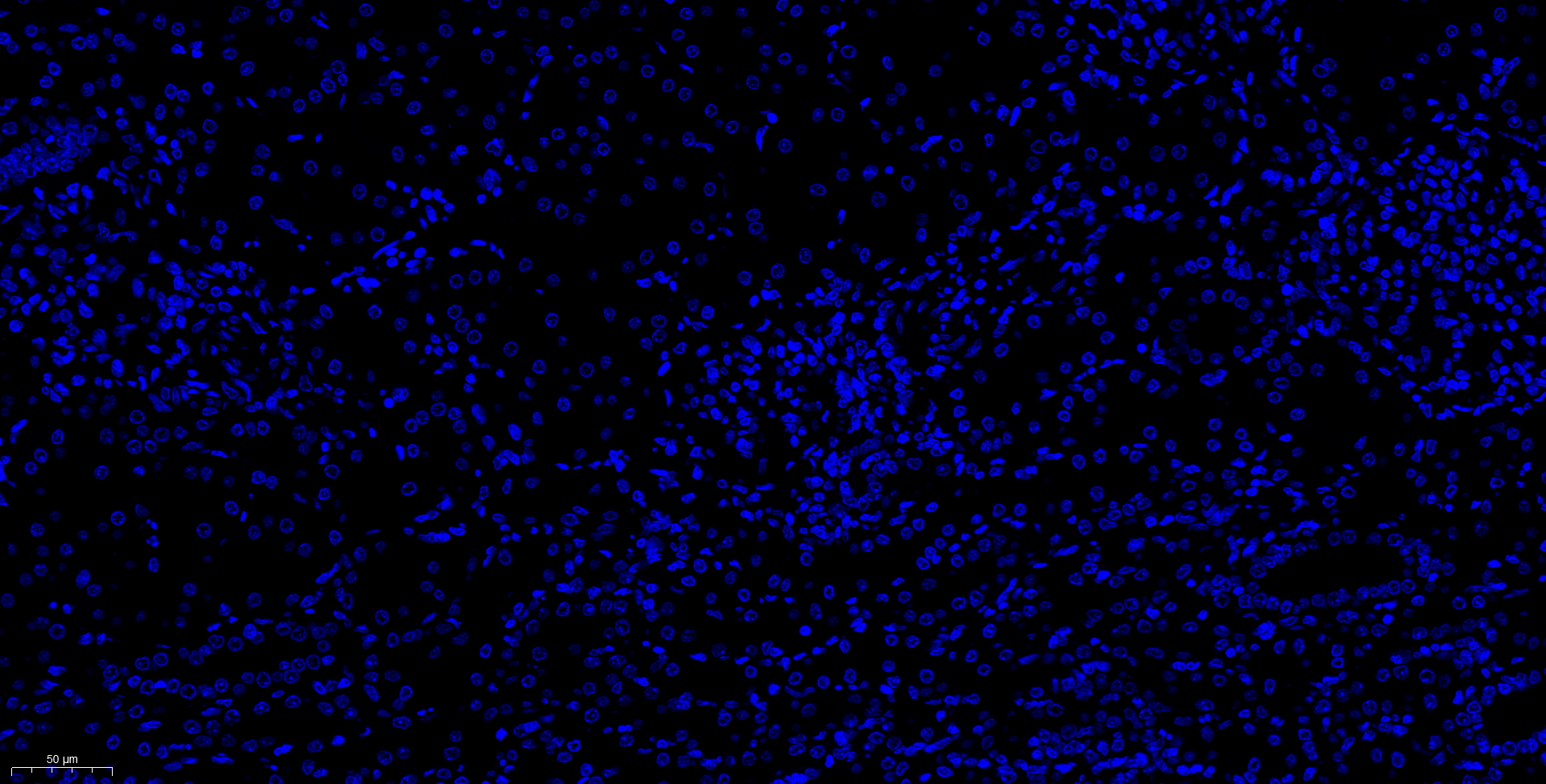

Supplement: Supplementary file 1 [file vetsci-12-00130-s001.zip › TEM、HE and IHC/Fig3-D4.jpg]

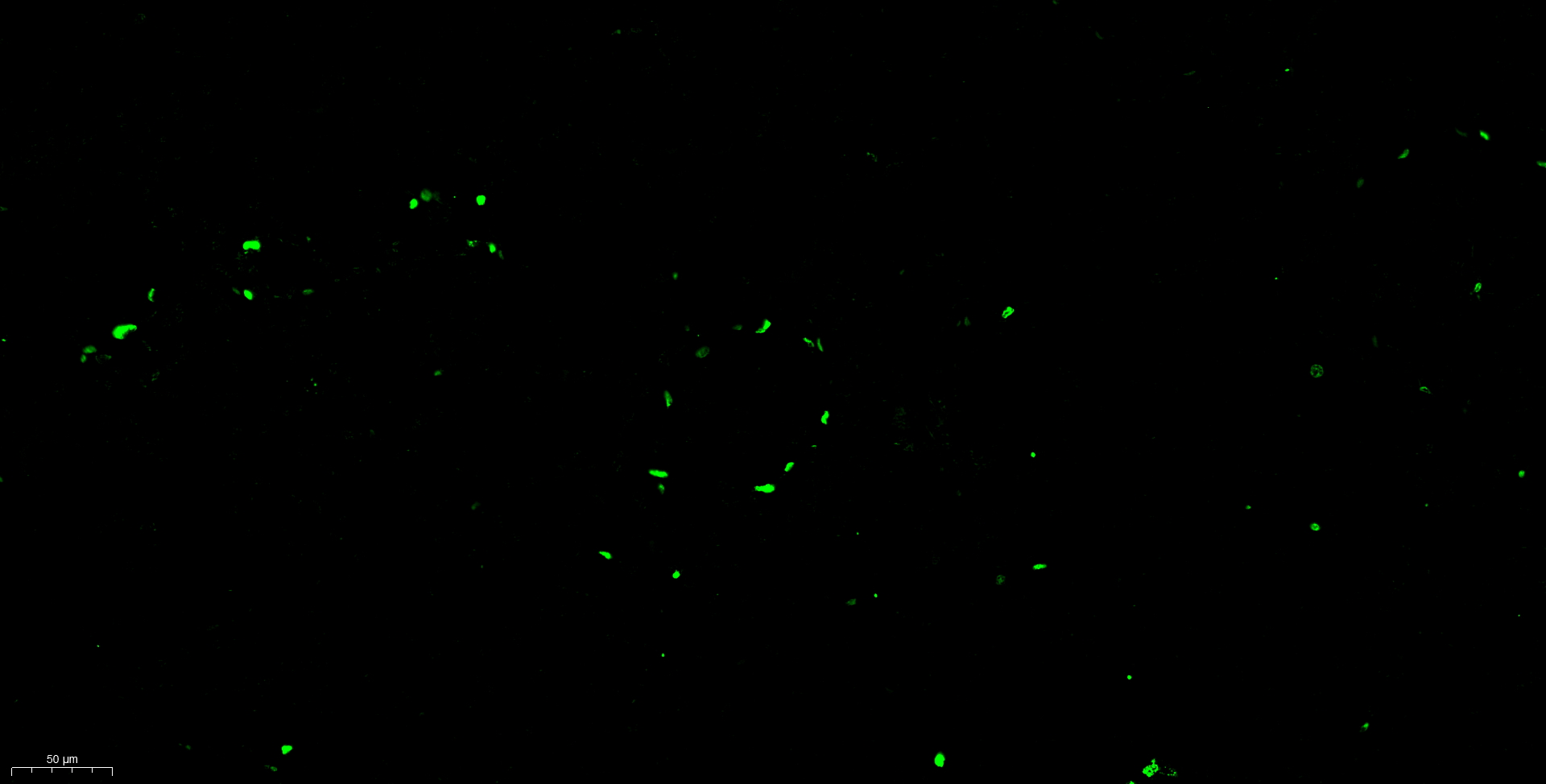

Supplement: Supplementary file 1 [file vetsci-12-00130-s001.zip › TEM、HE and IHC/Fig3-D5.jpg]

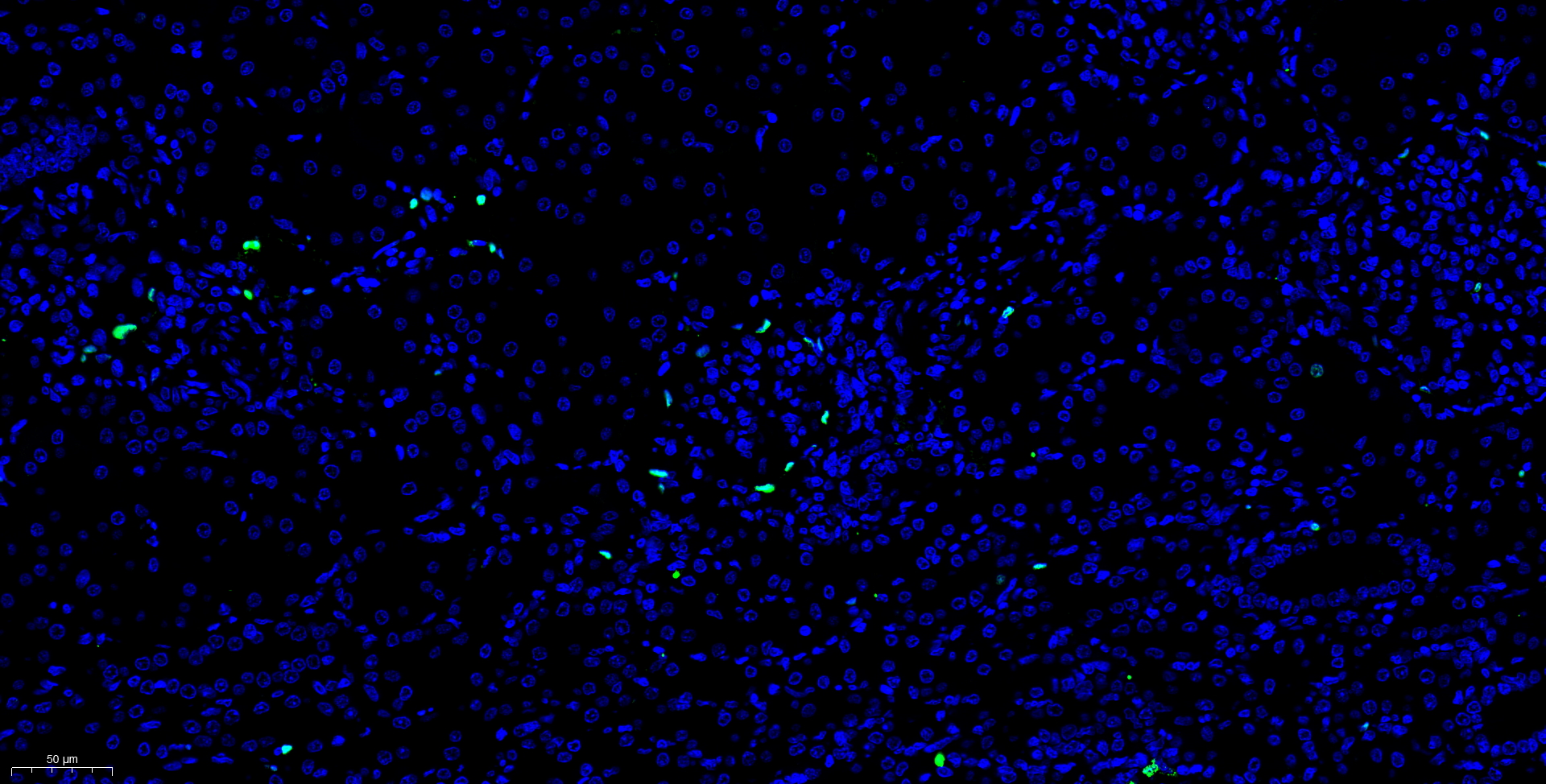

Supplement: Supplementary file 1 [file vetsci-12-00130-s001.zip › TEM、HE and IHC/Fig3-D6.jpg]

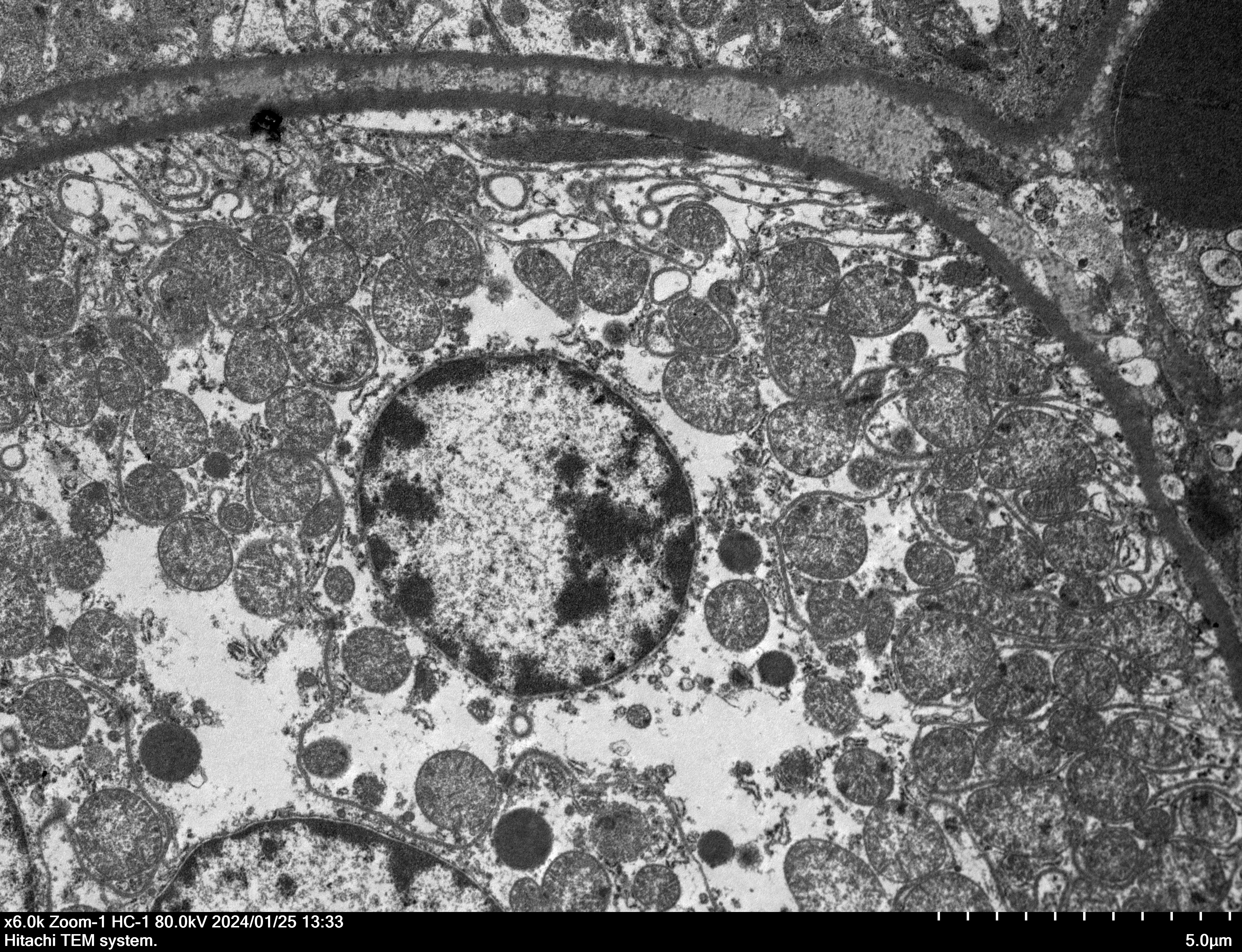

Supplement: Supplementary file 1 [file vetsci-12-00130-s001.zip › TEM、HE and IHC/Fig4-A.tif]

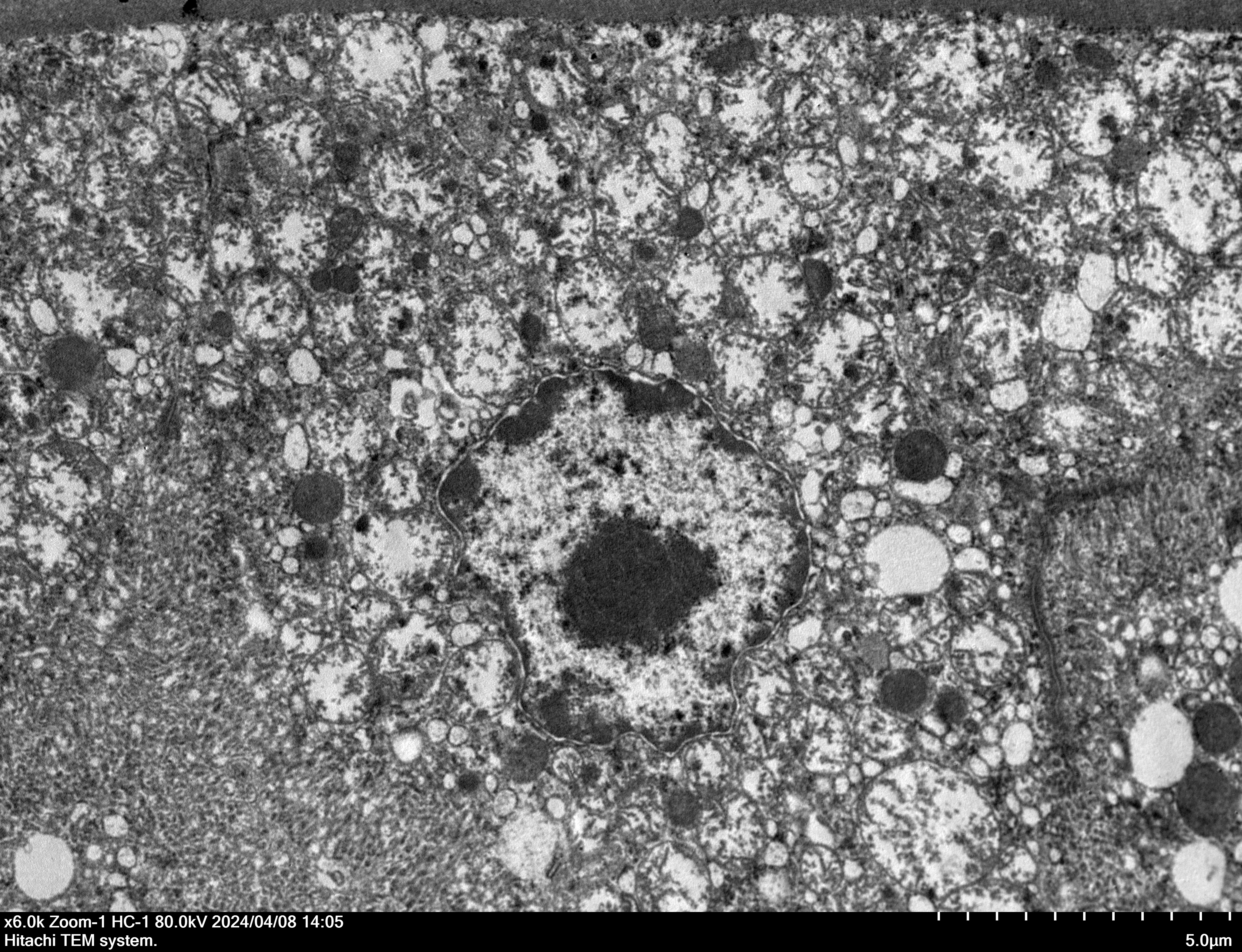

Supplement: Supplementary file 1 [file vetsci-12-00130-s001.zip › TEM、HE and IHC/Fig4-B.tif]

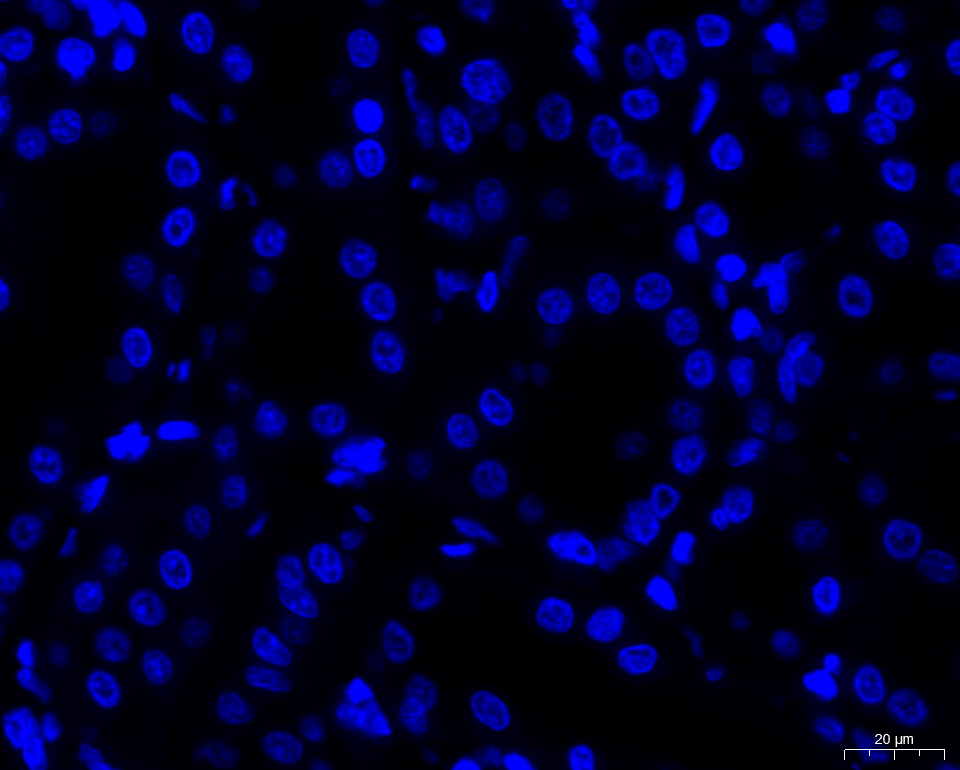

Supplement: Supplementary file 1 [file vetsci-12-00130-s001.zip › TEM、HE and IHC/Fig6-A1.jpg]

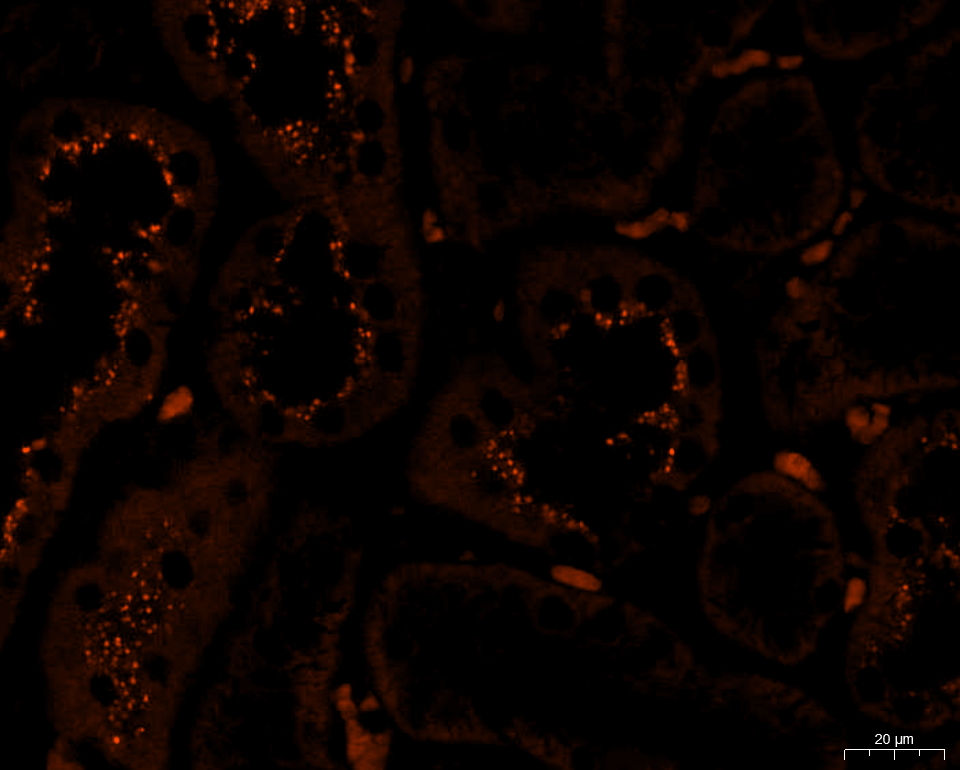

Supplement: Supplementary file 1 [file vetsci-12-00130-s001.zip › TEM、HE and IHC/Fig6-A2.jpg]

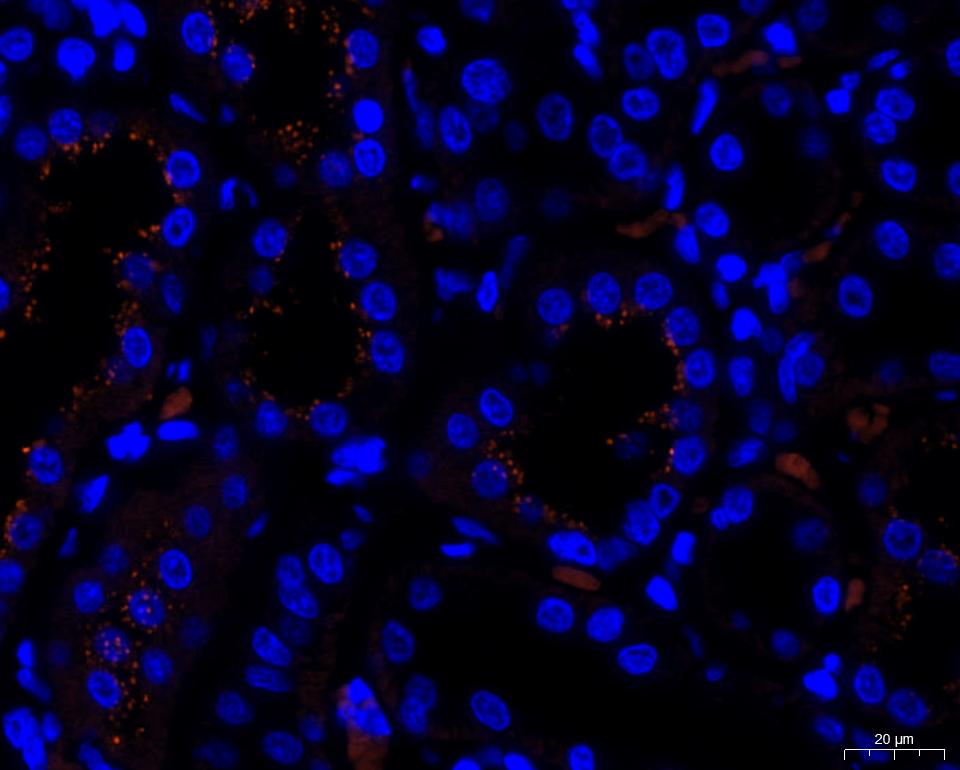

Supplement: Supplementary file 1 [file vetsci-12-00130-s001.zip › TEM、HE and IHC/Fig6-A3.jpg]

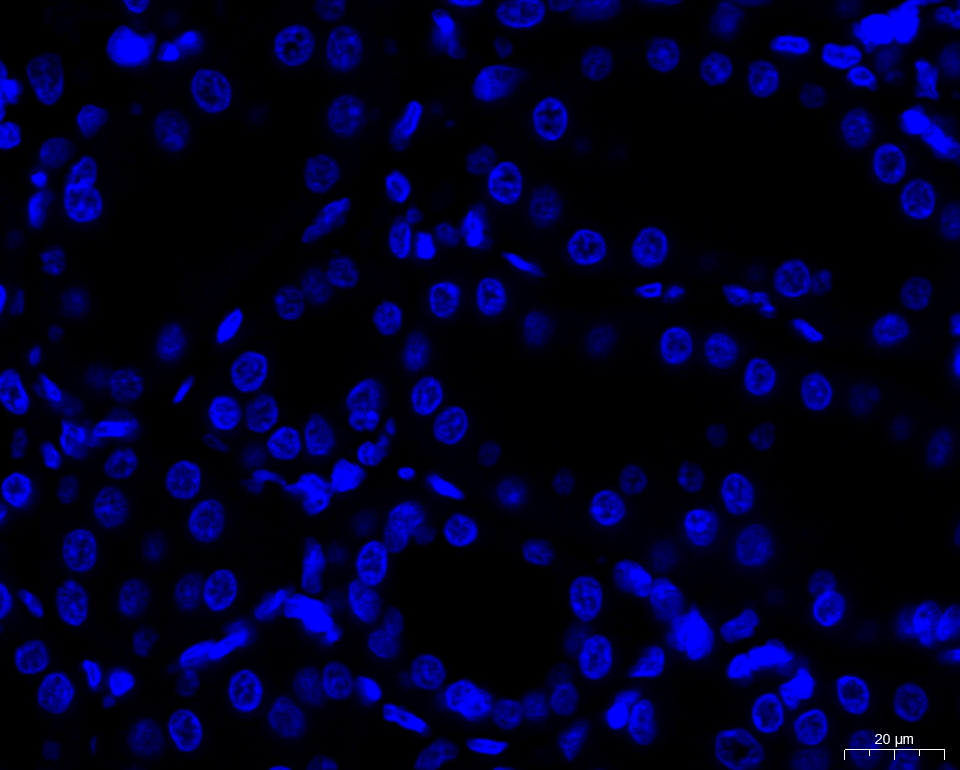

Supplement: Supplementary file 1 [file vetsci-12-00130-s001.zip › TEM、HE and IHC/Fig6-A4.jpg]

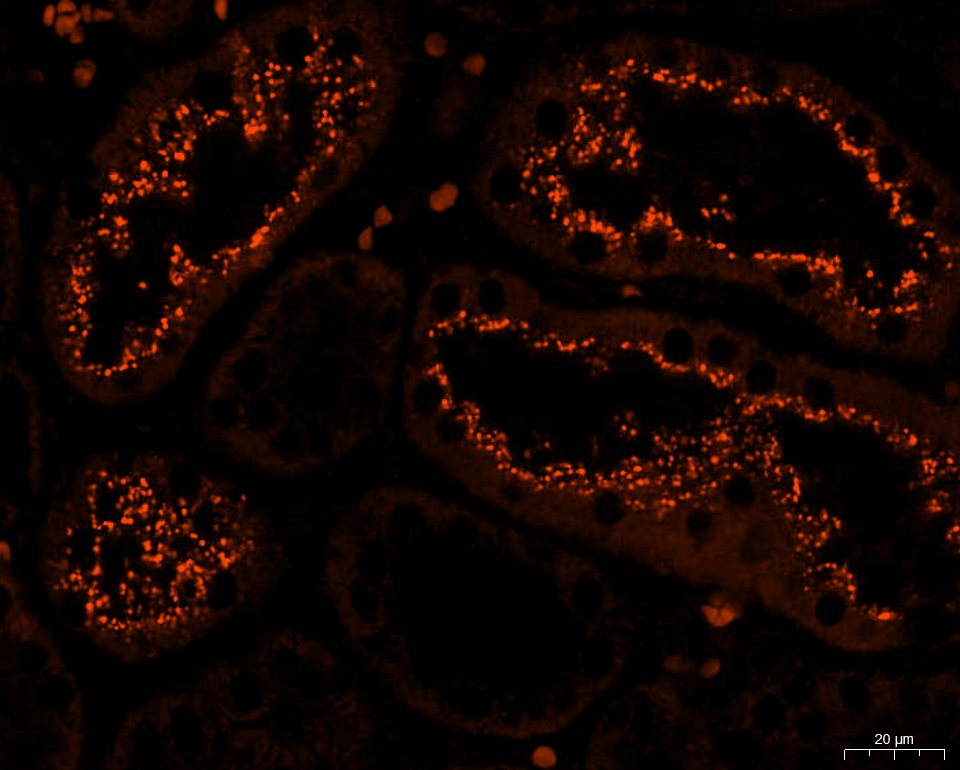

Supplement: Supplementary file 1 [file vetsci-12-00130-s001.zip › TEM、HE and IHC/Fig6-A5.jpg]

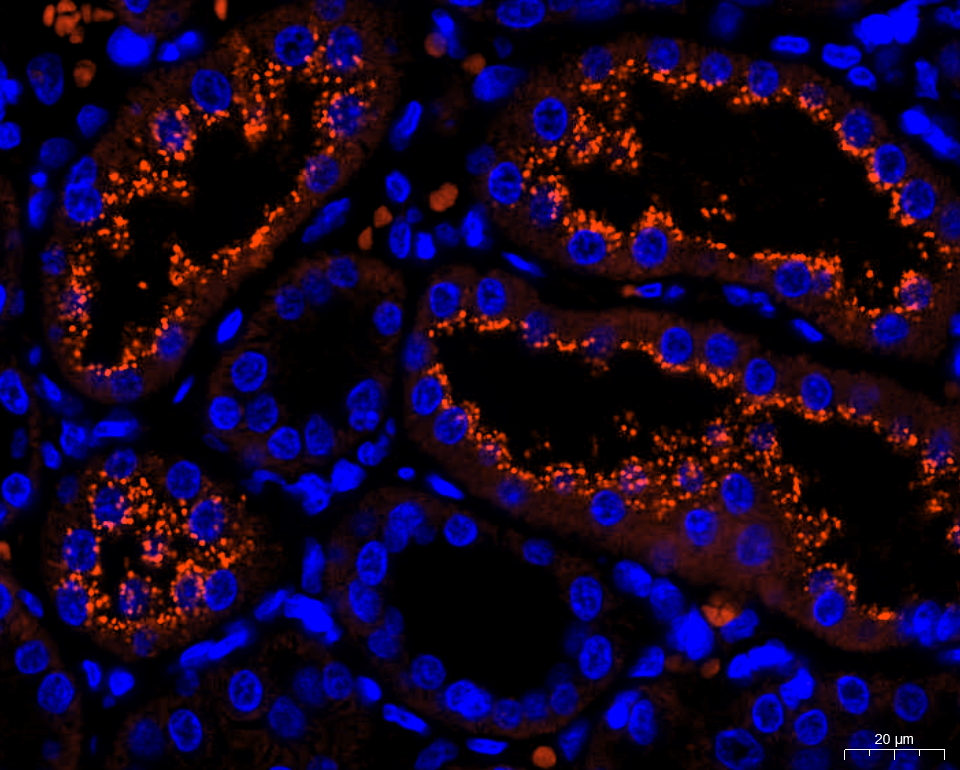

Supplement: Supplementary file 1 [file vetsci-12-00130-s001.zip › TEM、HE and IHC/Fig6-A6.jpg]

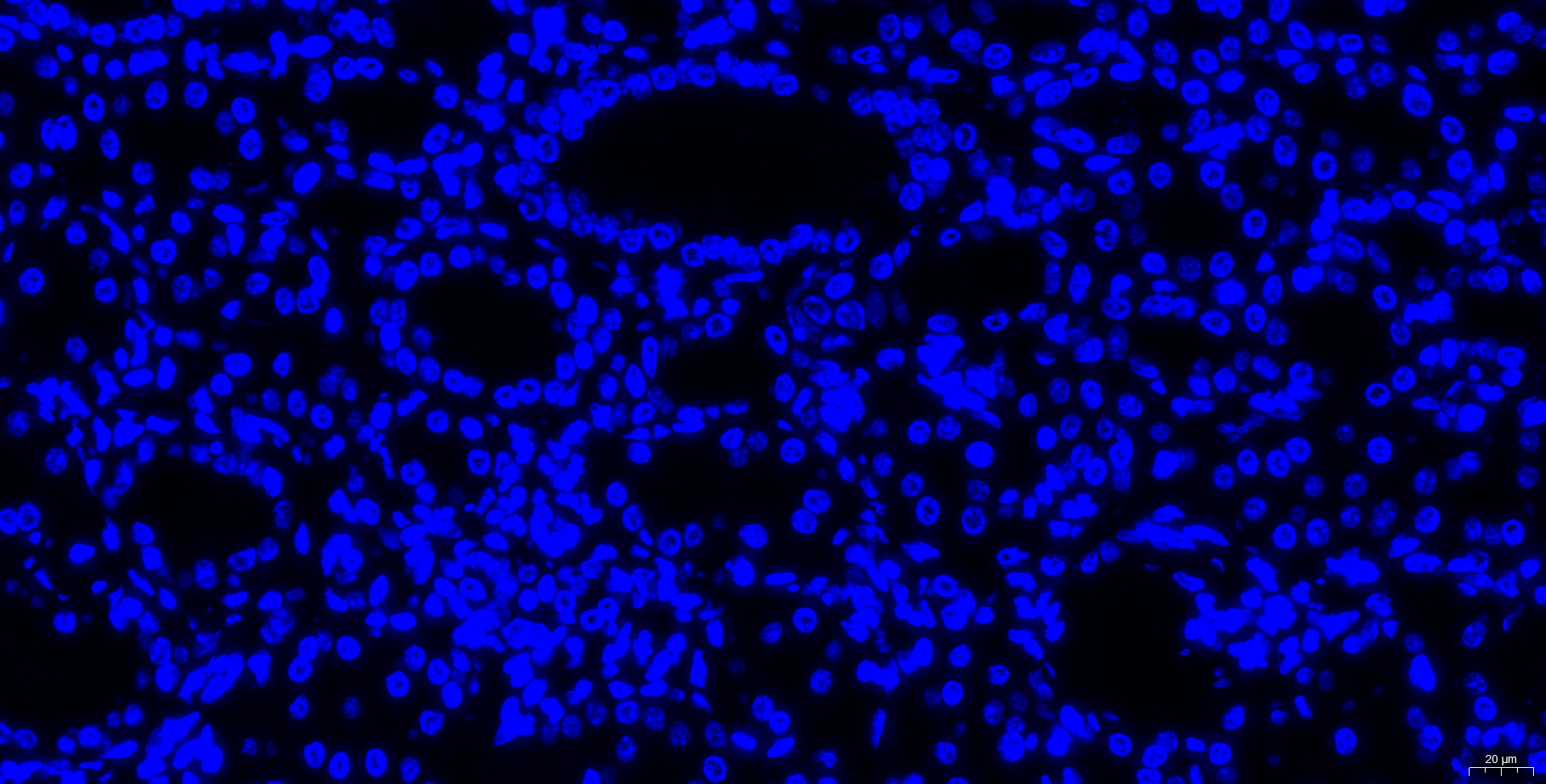

Supplement: Supplementary file 1 [file vetsci-12-00130-s001.zip › TEM、HE and IHC/Fig6-E1.jpg]

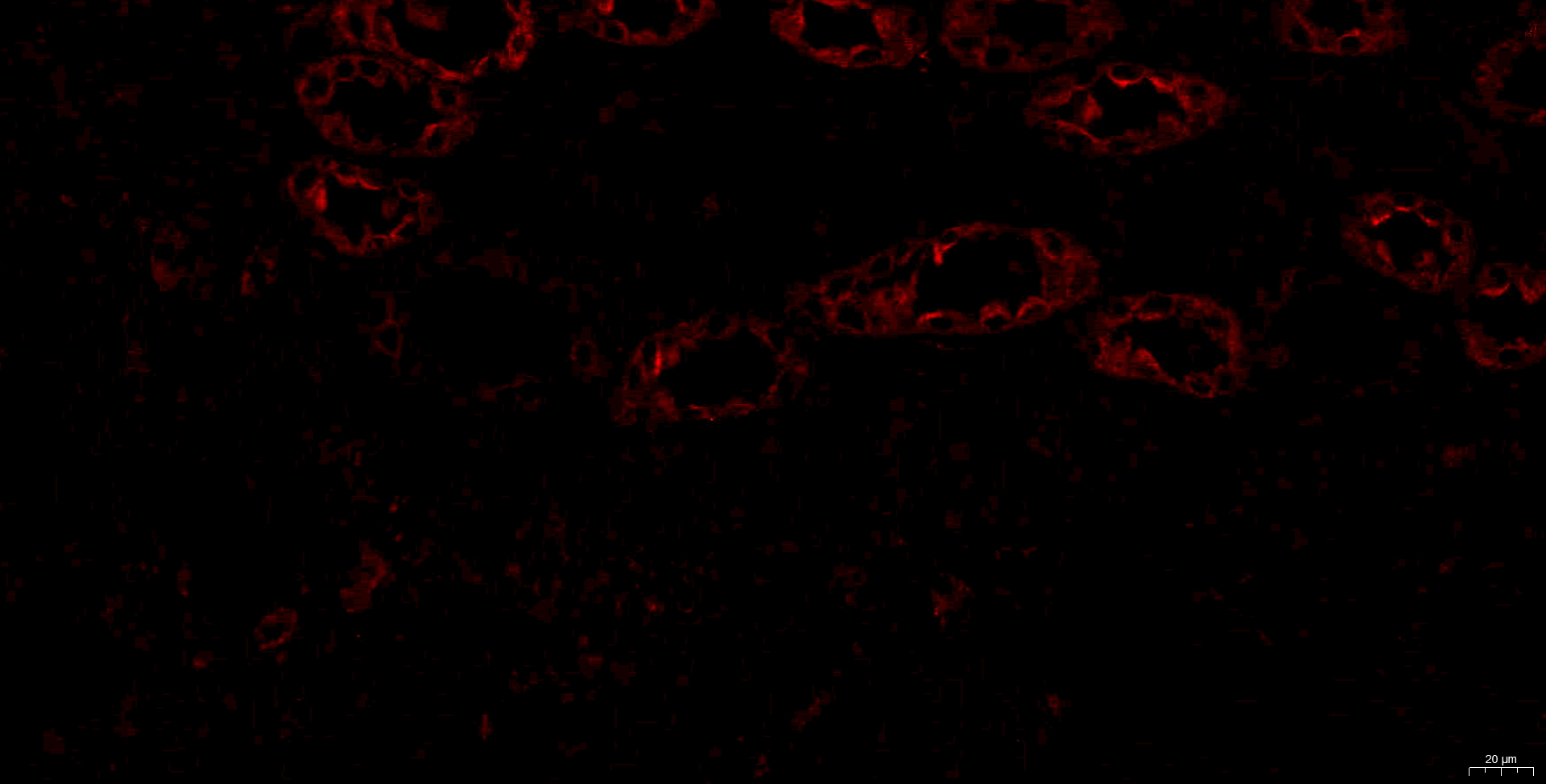

Supplement: Supplementary file 1 [file vetsci-12-00130-s001.zip › TEM、HE and IHC/Fig6-E2.jpg]

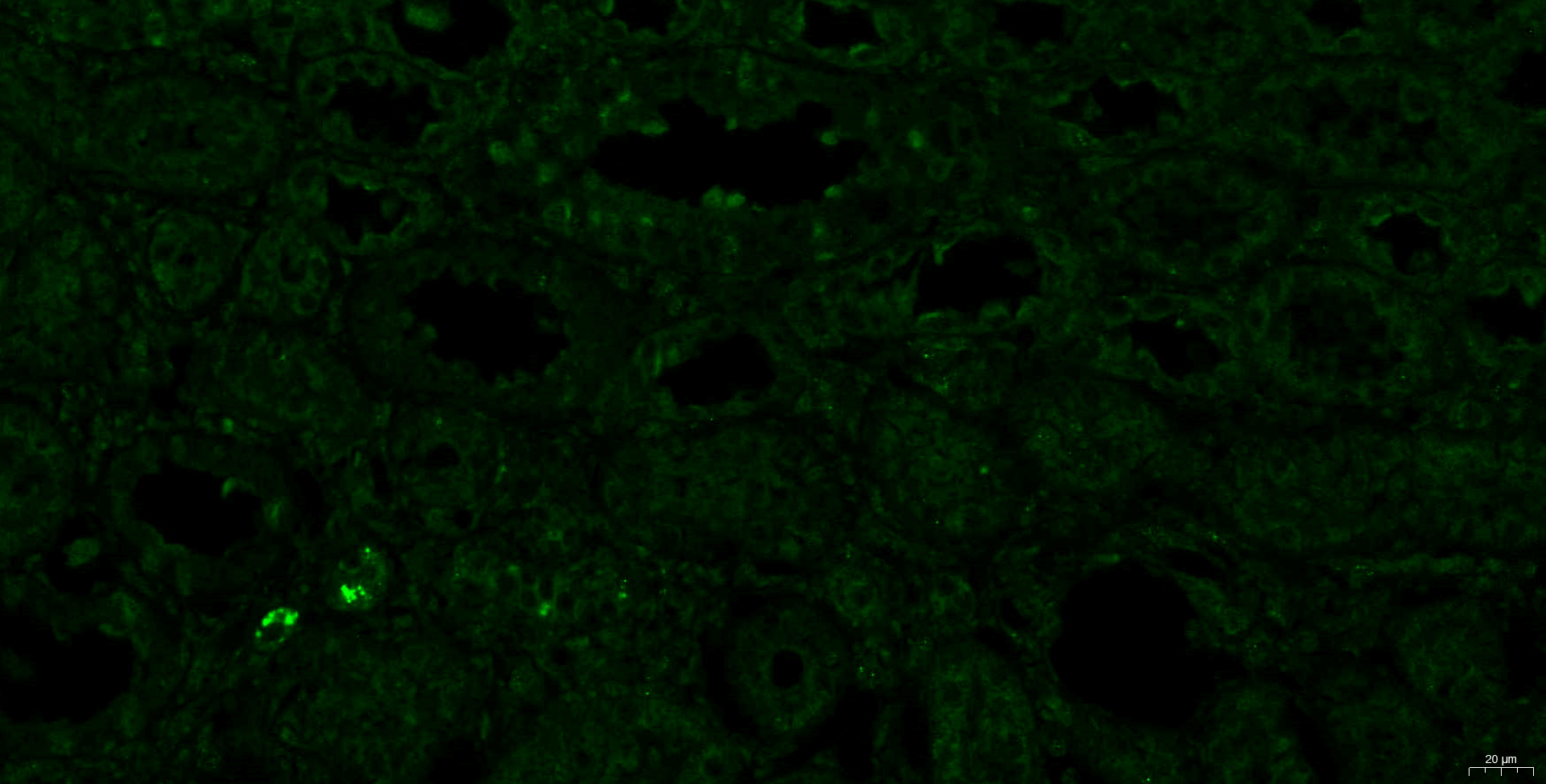

Supplement: Supplementary file 1 [file vetsci-12-00130-s001.zip › TEM、HE and IHC/Fig6-E3.jpg]

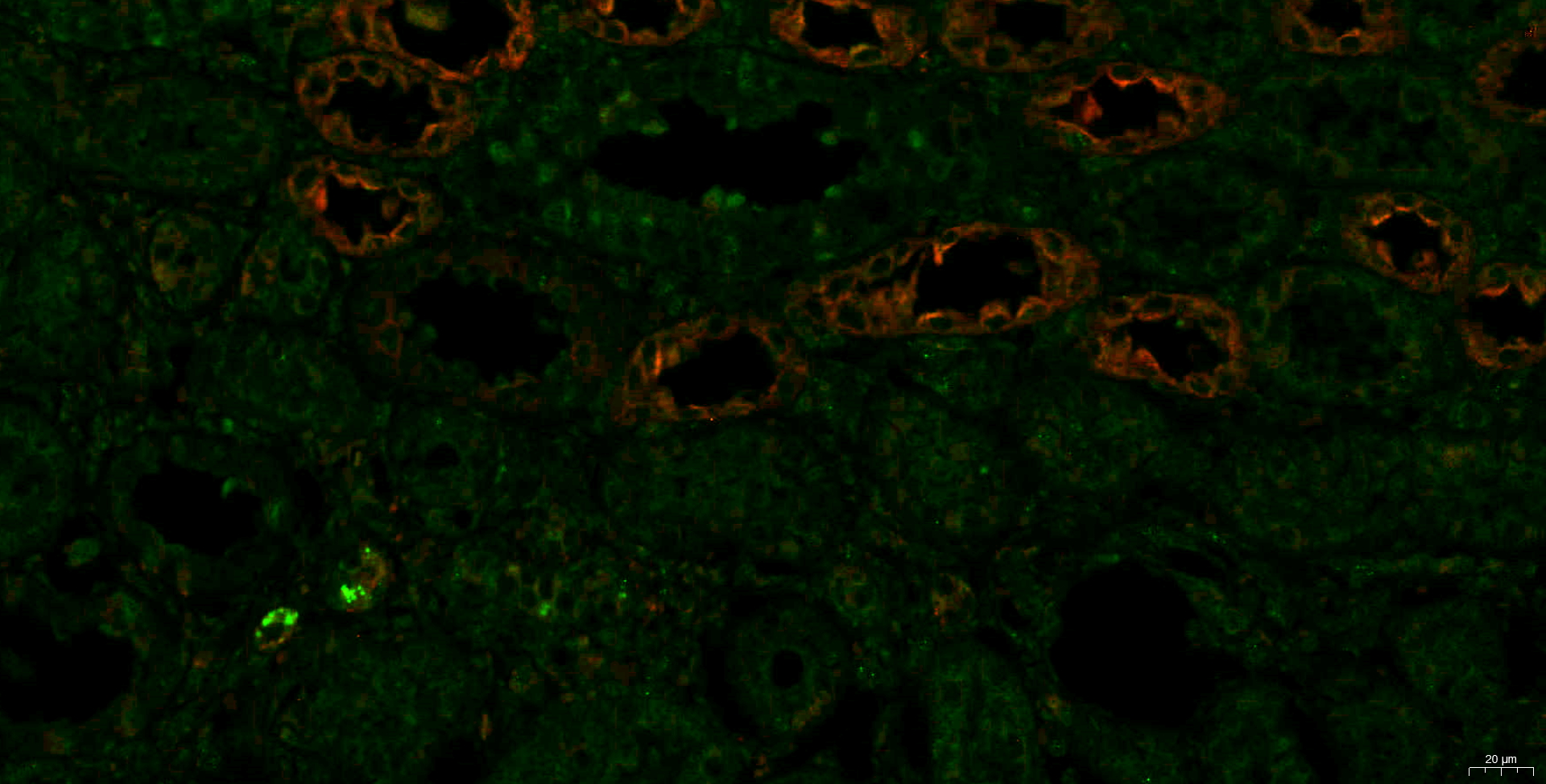

Supplement: Supplementary file 1 [file vetsci-12-00130-s001.zip › TEM、HE and IHC/Fig6-E4.jpg]

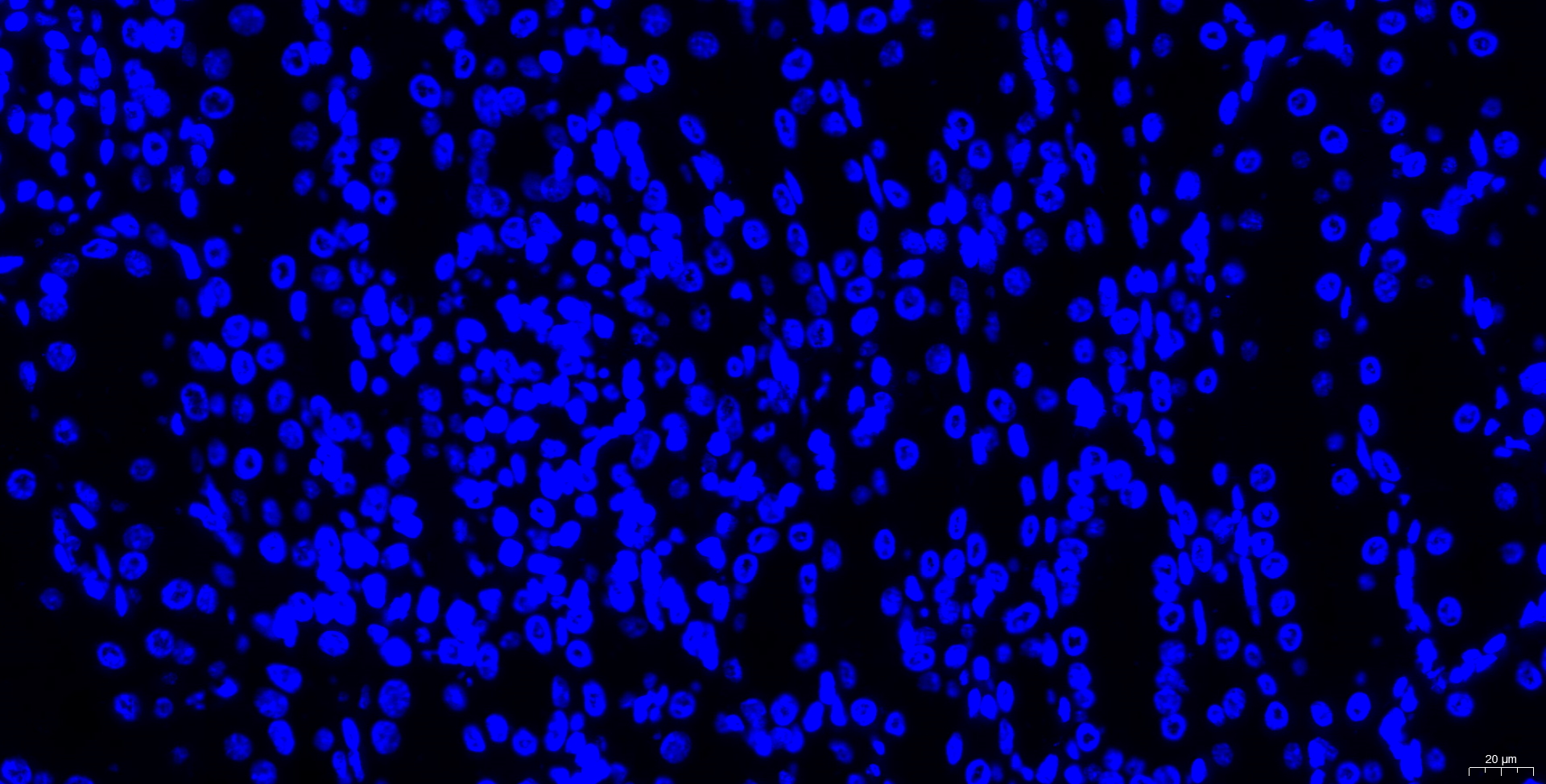

Supplement: Supplementary file 1 [file vetsci-12-00130-s001.zip › TEM、HE and IHC/Fig6-E5.jpg]

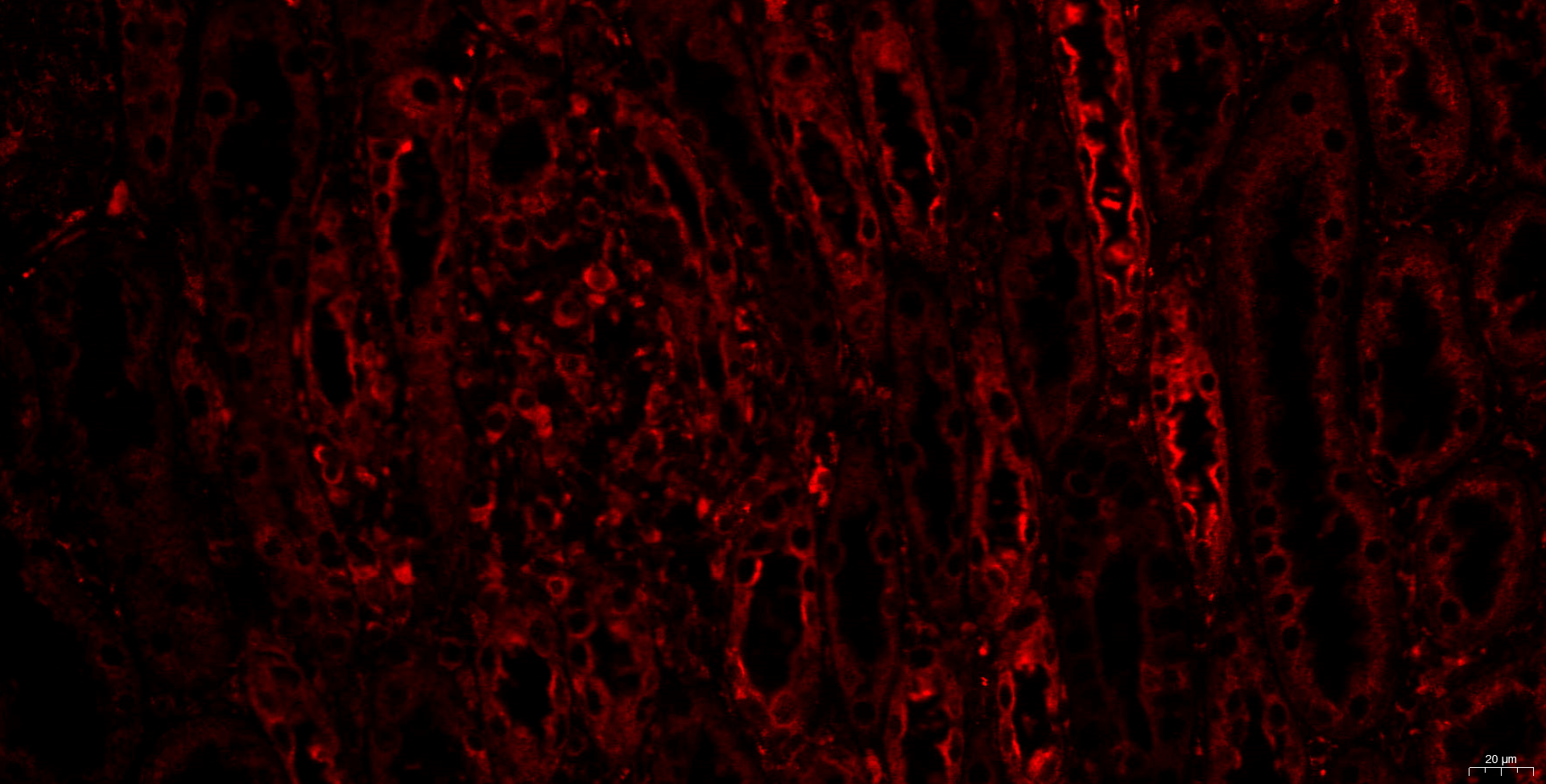

Supplement: Supplementary file 1 [file vetsci-12-00130-s001.zip › TEM、HE and IHC/Fig6-E6.jpg]

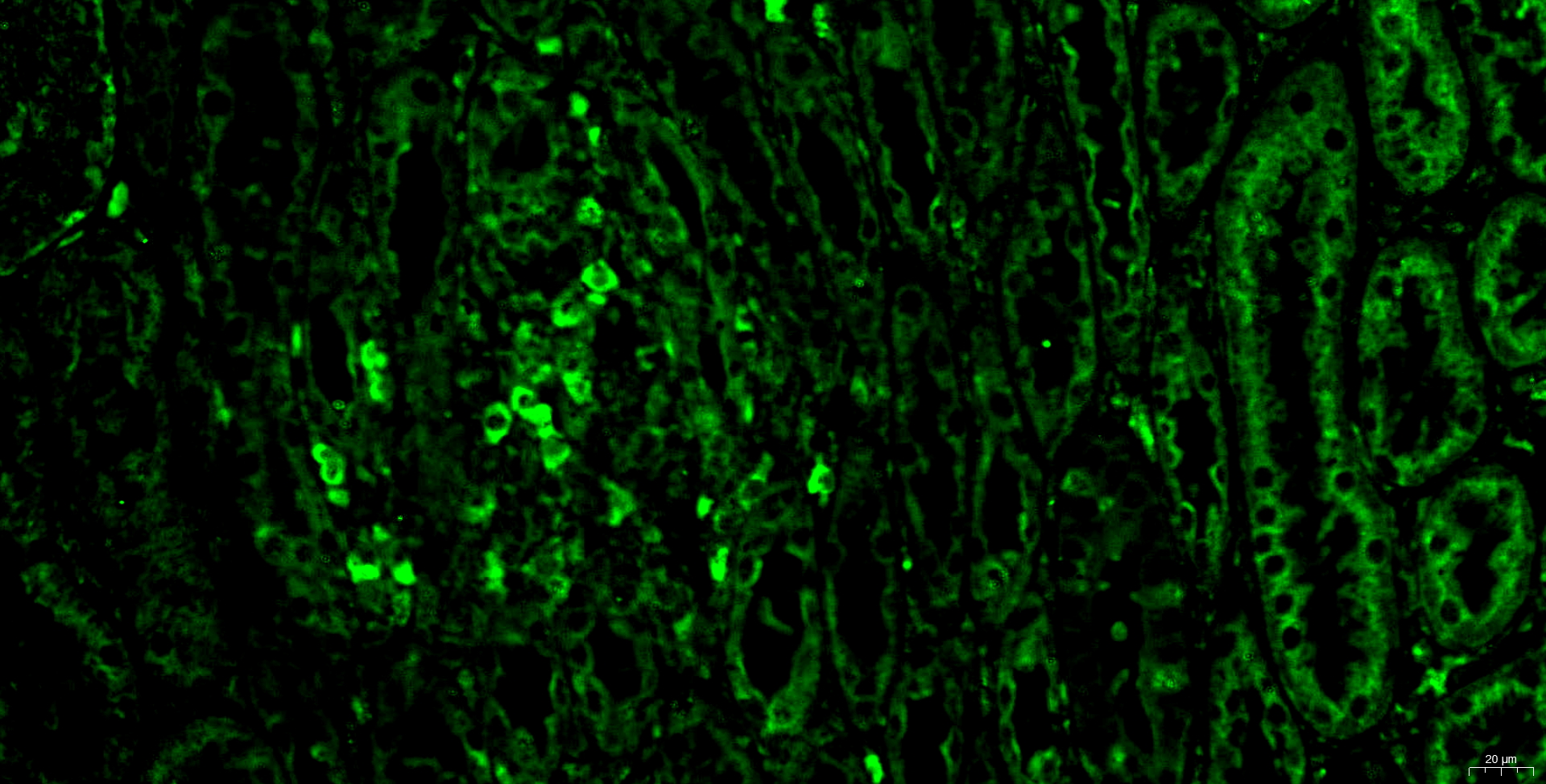

Supplement: Supplementary file 1 [file vetsci-12-00130-s001.zip › TEM、HE and IHC/Fig6-E7.jpg]

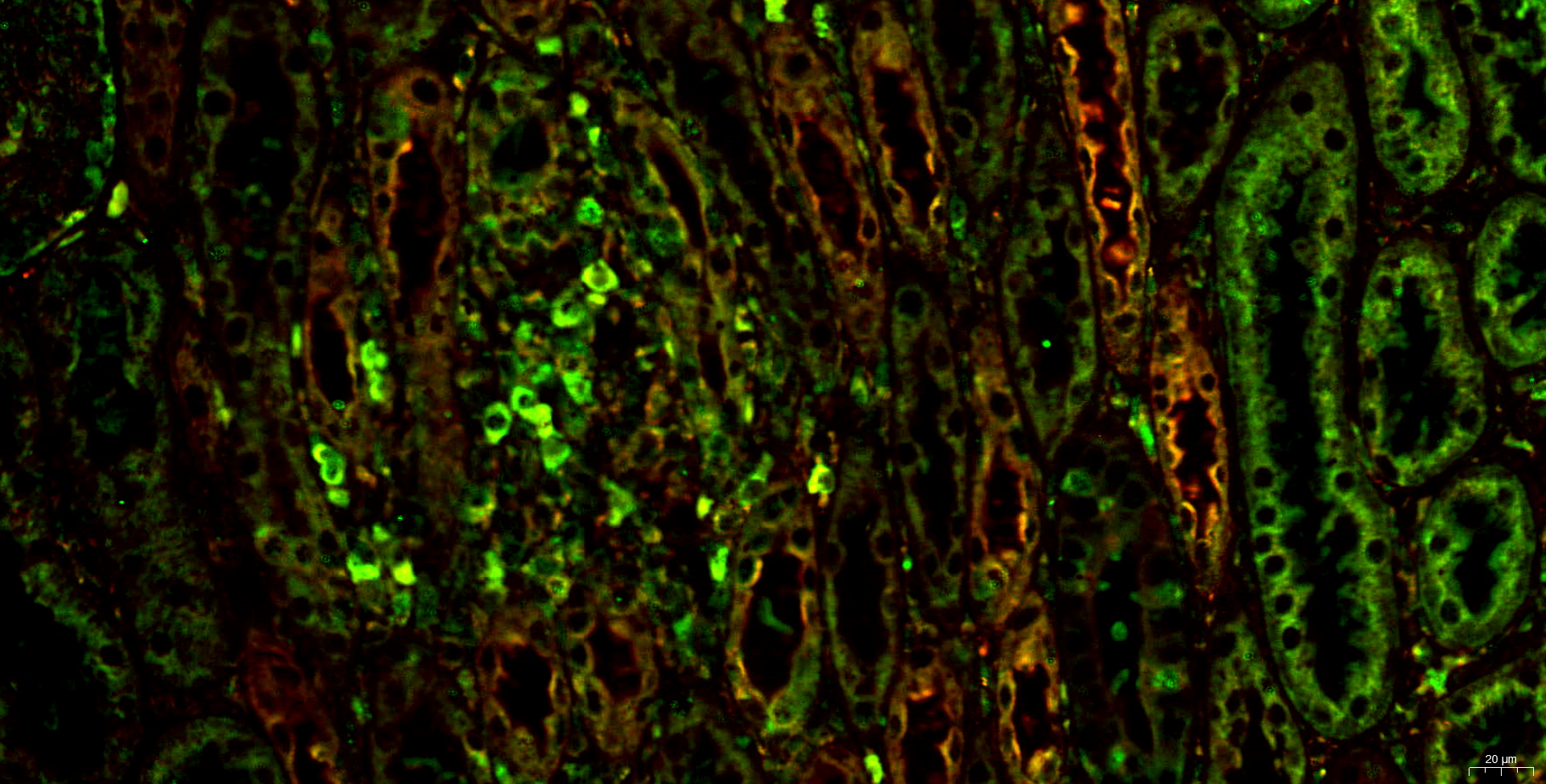

Supplement: Supplementary file 1 [file vetsci-12-00130-s001.zip › TEM、HE and IHC/Fig6-E8.jpg]
